# Causal evidence for a coordinated temporal interplay within the language network

**DOI:** 10.1073/pnas.2306279120

**Published:** 2023-11-14

**Authors:** Joëlle A. M. Schroën, Thomas C. Gunter, Ole Numssen, Leon O. H. Kroczek, Gesa Hartwigsen, Angela D. Friederici

**Affiliations:** ^a^Department of Neuropsychology, Max Planck Institute for Human Cognitive and Brain Sciences, Leipzig 04103, Germany; ^b^Methods and Development Group Brain Networks, Max Planck Institute for Human Cognitive and Brain Sciences, Leipzig 04103, Germany; ^c^Lise Meitner Research Group Cognition and Plasticity, Max Planck Institute for Human Cognitive and Brain Sciences, Leipzig 04103, Germany; ^d^Department of Psychology, Clinical Psychology and Psychotherapy, Universität Regensburg, Regensburg 93053, Germany; ^e^Cognitive and Biological Psychology, Wilhelm Wundt Institute for Psychology, Leipzig 04109, Germany

**Keywords:** brain dynamics, language network, N400, TMS-EEG

## Abstract

Recent neurobiological models on language suggest that auditory sentence comprehension is supported by a coordinated temporal interplay within a left-dominant brain network, including the posterior inferior frontal gyrus (pIFG), posterior superior temporal gyrus and sulcus (pSTG/STS), and angular gyrus (AG). Here, we probed the timing and causal relevance of the interplay between these regions by means of concurrent transcranial magnetic stimulation and electroencephalography (TMS-EEG). Our TMS-EEG experiments reveal region- and time-specific causal evidence for a bidirectional information flow from left pSTG/STS to left pIFG and back during auditory sentence processing. Adapting a condition-and-perturb approach, our findings further suggest that the left pSTG/STS can be supported by the left AG in a state-dependent manner.

## Significance

Language is efficiently processed in milliseconds in the human brain. It is supported by the interaction of widely distributed brain regions in the left frontal, temporal, and parietal cortex, which are interconnected via white matter fiber tracts, allowing fast information transfer between them. The precise timing of this interaction within the language network is still an open question as causal evidence is insufficient. The present three experiments used a combined transcranial magnetic stimulation and electroencephalography approach to provide causal evidence for region-specific, time-critical processing-windows during auditory language comprehension. Our results show that a temporally well-coordinated interaction of left posterior temporal and inferior frontal regions provides the basis for the human ability to process language fast and efficiently.

During everyday conversation, listeners need to rapidly and efficiently extract different types of information from the speech signal (i.e., phonological, syntactic, semantic) to understand the communicated message. This complex process is supported by the interaction between widely distributed regions of a left-dominant fronto-temporo-parietal network, taking place on the order of milliseconds. In the last decade, a large number of models on the neurobiology of language theorized about the information flow within this network (1–8). Based on a rich body of neuroimaging and electrophysiological studies reviewed in ref. 9, the current proposal is that information is first analyzed in posterior temporal brain regions and then mapped to the prefrontal cortex. Prefrontal projections back to the posterior temporal cortex are suggested to subserve subsequent top-down processes see also ref. 10. Several magnetoencephalography (MEG) and intracranial electroencephalography (EEG) studies have provided correlational evidence for the spatiotemporal dynamics of sentence comprehension (11–17). Effective connectivity analyses further supported the assumed information flow (18–22). As a next step toward plausible neurobiological models of language, a more detailed and causal link (23–25) between the time and place of speech comprehension in the brain is wanted. To date, however, causal evidence for the precise timing of the interplay within the language network (where, what, and when) is still lacking.

As a step toward addressing this research gap, we combined transcranial magnetic stimulation (TMS) with concurrent EEG measurements. TMS is a noninvasive brain stimulation method that can manipulate brain activation at precise points in time (26). When combined with simultaneous EEG, TMS becomes a sophisticated method (TMS-EEG) capable of providing causal information about brain dynamics underlying, for instance, auditory language comprehension. Across three distinct TMS-EEG experiments, we systemically varied the timing of triple-pulse (10 Hz) online repetitive TMS (rTMS) to test when neural activity in the left posterior inferior frontal gyrus (pIFG) and posterior superior temporal gyrus and sulcus (pSTG/STS) is causally relevant for auditory sentence processing. During short sentences (i.e., pronoun–verb–article–noun), online rTMS was applied during three distinct time-windows relative to verb onset, either testing early (0 to 200 ms), intermediate (150 to 350 ms), or late (300 to 500 ms) processing windows. Given the presence of extremely large artifacts in the EEG signal following TMS over lateral brain regions (27–30), we utilized the large and robust N400 effect at the sentence-final noun (31, 32) as a read-out to draw inferences about the causal relevance of the left pIFG and pSTG/STS during the earlier verb processing phase.

In explaining the logic of our experimental design, we adopted a well-established N400 paradigm used in several earlier studies (33–36). In this sentence paradigm (e.g., *He drinks the beer*), verbs play a major role by setting prior restrictions on which semantic features the noun argument is expected to have (e.g., *drinks* requires a “liquid” object). The preactivation of these features has been linked to the so-called reversed N400 effect (35, 36), as reflected by a larger N400(m) response for highly (e.g., * drinks*) versus less predictive verbs (e.g., *sees*). The benefit of this preactivation is indicated by the classical N400 effect at the subsequent noun position (31, 37, 38), with a smaller N400(m) response evoked by expected nouns (e.g., *He drinks the beer*) compared to a situation where the same noun is less expected (e.g., *He sees the beer*). Thus, if a brain region is causally relevant for a specific verb processing phase, its rTMS perturbation leads to downstream effects observable at the noun position.

Implementing a similar experimental design, a recent TMS-EEG study (36) showed, however, that triple-pulse rTMS (10 Hz) over left pIFG or pSTG/STS at verb onset (early time-window) only results in short-living modulation of event-related potentials (ERPs), i.e., a perturbation effect. In fact, perturbation of only one node in the wide-spread fronto-temporo-parietal language network more often appears insufficient to temporally disrupt processing (36, 39, 40), indicating a high degree of compensation and flexible adaptation within the language system (41, 42).

Evidence suggests that robustness against unifocal rTMS perturbation can be effectively influenced by increasing the overall perturbation load on the system (39, 42). Therefore, offline continuous theta burst stimulation [cTBS, (43)] was applied to another critical node within the same hemispheric network prior to targeting the left pIFG or pSTG/STS with online rTMS (i.e., the so-called “condition-and-perturb” approach). Given that the distinct components of language are systematically connected (44), it is reasonable to assume that the contribution of a particular brain region likely depends on the interaction with other regions of this large-scale language network (45).

In the present set of experiments, we decided to always target the left angular gyrus (AG) with cTBS due to its supporting role in sentence comprehension. This decision was mainly based on an earlier condition-and-perturb study (39) showing that AG-cTBS can effectively sensitize the language system to subsequent online rTMS over a prefrontal network node. In addition, several reviews and meta-analyses consistently linked activation of the left AG to semantic processing (10, 46–49), and this region has also been implicated in deficits involving sentence comprehension (50). A functional magnetic resonance imaging (fMRI) study (34) using similar sentence materials as in our study also showed that left AG activation accompanies successful speech comprehension. Further evidence from TMS confirmed that this brain region is causally relevant for speech comprehension (51). Finally, the left AG is densely interconnected with the rest of the core language network (46, 52, 53), thereby supporting interactions with other parts of the language system. Given these findings, we assumed that left AG-cTBS would pave the way for an online rTMS effect of left pIFG and/or left pSTG/STS in the current study.

**Fig. 1.**
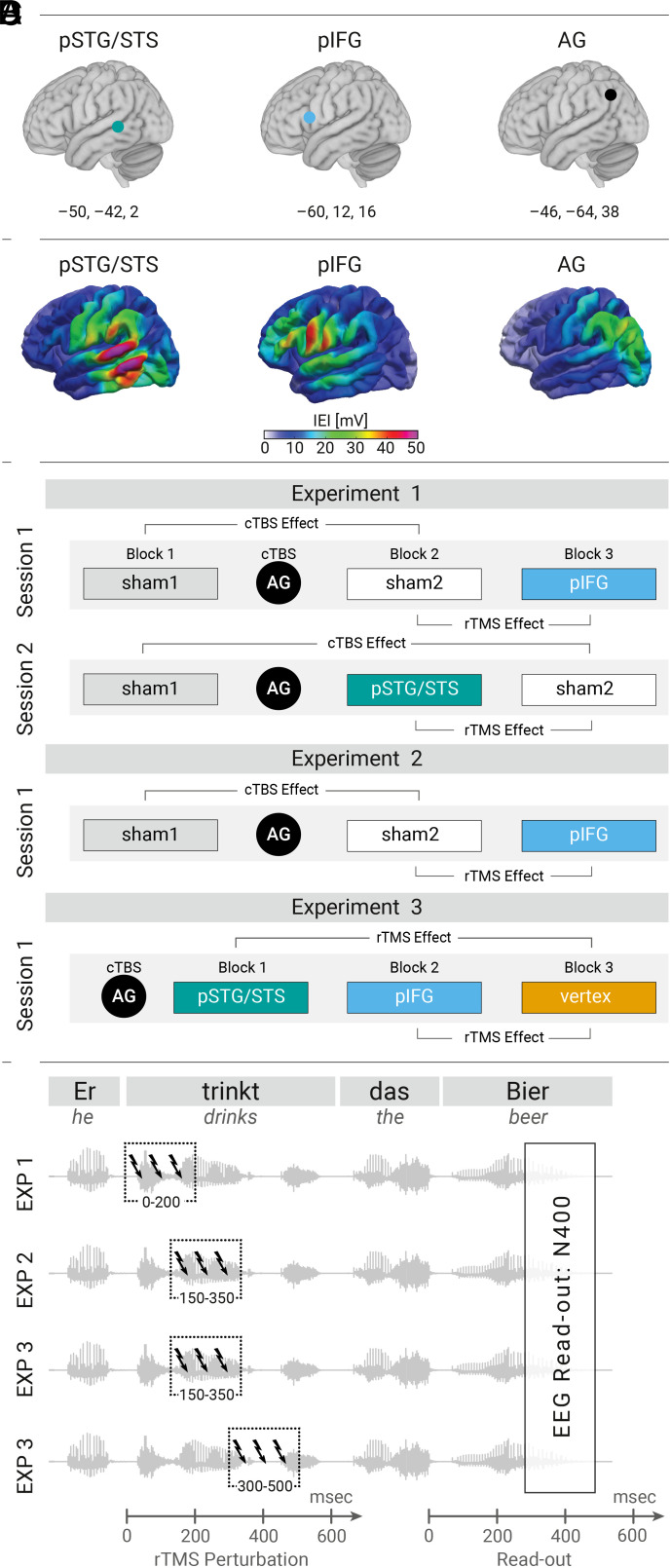
Overview of the experimental design. (*A*) Targets and their MNI coordinates (x, y, z). (*B*) Simulations of the induced electric fields by TMS verified effective stimulation exposure. Left pSTG/STS and pIFG were stimulated with 90% RMT and AG with 80% RMT. Color: Group-averaged magnitude |E| induced e-field in mV for Experiment 1. (*C*) Offline cTBS (AG) was combined with online rTMS (pIFG, pSTG/STS, vertex, or sham). cTBS effect: comparison of the ineffective blocks before (Sham1) and after cTBS (Sham2). rTMS effects: comparison of the effective target (pIFG or pSTG/STS) against control (Exp.1 & 2: sham2, Exp.3: vertex). The order of the blocks following cTBS was counterbalanced between participants. (*D*) 3-pulse online rTMS (10 Hz) was applied during one of three time-windows relative to the verb position. The N400 effect at the noun position was our read-out for inferring causality. MNI, Montreal Neurological Institute; cTBS, continuous theta burst stimulation; rTMS, repetitive transcranial magnetic stimulation; RMT, resting motor threshold.

Based on the earlier-mentioned information flow within the language network pointed out in the literature (3, 9), we hypothesized a temporal dissociation of the causal involvement of the left pIFG and pSTG/STS. More specifically, we expected that the left pSTG/STS would show causal relevance during the early time-window (Experiment 1), as this region has been shown to support the initial auditory perception of words (54, 55). Based on MEG evidence hinting at the subsequent involvement of the prefrontal cortex (11, 12, 17, 56), causal relevance of the left pIFG was expected during the intermediate time-window (Experiments 2 and 3). If further top-down processing from the prefrontal back to the temporal cortex takes place (3, 9), we expected to observe causal relevance of the left pSTG/STS again during the late time-window (Experiment 3). An overview of the online rTMS perturbation conditions per experiment is visualized in Fig. 1.

## Results and Discussion

Our experimental designs (Fig. 1*C*) allowed us to address several specific research questions of interest, as discussed below. For each planned comparison, we report the results of a series of linear mixed-effect regression models (see *EEG Statistical Analysis* for more details). For the sake of clarity, not all statistical details are reported here. Full model summaries can be found in *SI Appendix*, Tables S4–S21.

### Verification of Effective Stimulation.

The strength and spread of the electric field induced by TMS (e-field) depends on various factors (57), including TMS coil placement (58), stimulation intensity (59), individual gyrification patterns (60), and the distribution of tissue types (61). Therefore, post hoc e-field simulations were conducted to verify effective stimulation of the cortical targets in all subjects. See Fig. 1*B* for group-averaged cortical e-fields for Experiment 1, and see *SI Appendix*, Fig. S1 for average and variance plots for all three experiments separately.

### Experiment 1.

Experiment 1 addressed two research questions. First, we included a block of ineffective sham rTMS before (Sham1) and after cTBS (Sham2) in order to examine whether cTBS of left AG alone modulates sentence-based semantic processing (i.e., cTBS effect, see Fig. 1*C*). Second, we probed the causal relevance of both left pIFG and pSTG/STS (after left AG-cTBS) during the early rTMS time-window (i.e., online rTMS effects). Here, the effective rTMS condition was compared to the ineffective condition after cTBS (Sham2) of that particular session (Fig. 1*C*). As discussed above, causal relevance of these brain regions during any of the three time-windows would be indicated by a modulation of the classical N400 effect at the noun position (Fig. 1*D*).

#### Effects of AG-cTBS on the N400.

The analysis of the noun position revealed a significant main effect of Semantic expectancy, with low cloze sentences evoking a greater negativity than high cloze sentences (i.e., classical N400 effect; *SI Appendix*, Fig. S1). This finding nicely replicates previous studies using similar sentence materials (33, 35, 36). A significant interaction of Semantic expectancy × Anteriority showed that this classical N400 effect was larger at posterior electrodes (β = −2.25, *SE* = 0.251, *t* = −8.954, and *P*< 0.001) compared to anterior electrodes (β = −1.71, *SE* = 0.251, *t* = −6.821, and *P*< 0.001), which fits with the usual N400 scalp distribution (62). In agreement with earlier TMS findings (39, 40), the classical N400 effect was not modulated by cTBS over left AG (i.e., no interaction with the TMS condition).

As an additional analysis, we also investigated the reversed N400 typically observed at the preceding midsentence verb. Replicating earlier studies (35, 36), this analysis indicated a main effect of Semantic predictability (β = −0.270, *SE* = 0.045, *t* = −5.960, and *P*< 0.001), with high predictive verbs eliciting a greater negativity than low predictive verbs (i.e., reversed N400 effect; *SI Appendix*, Fig. S1). Consistent with our findings at the noun position, however, this reversed N400 effect was not modulated by cTBS over the left AG.

#### Effects of early pSTG/STS-rTMS on the N400.

**Fig. 2.**
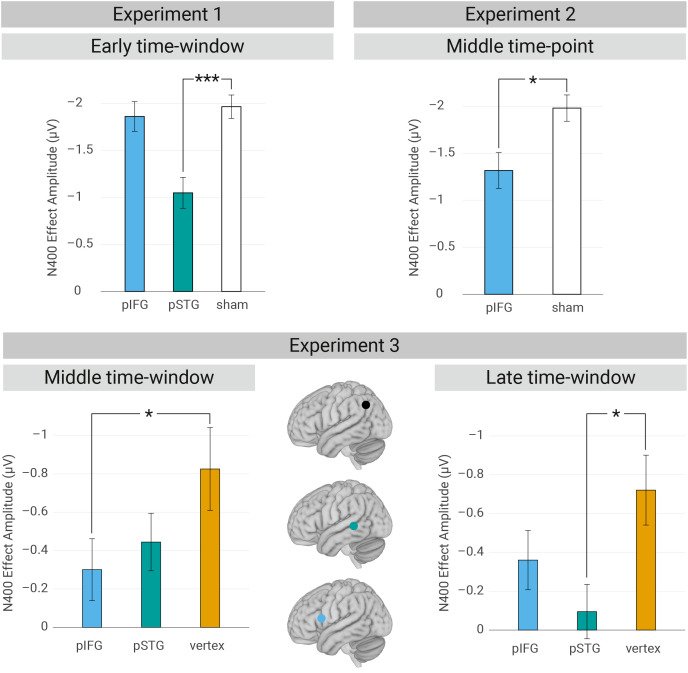
Overview of the rTMS perturbation effects. Mean classical N400 amplitude for each target region in the early, intermediate and late time-windows, with the N400 operationalized as the average voltage across all time points between 300 and 500 ms across all electrode sites within our ROI. Negative voltage is plotted upward. Error bars represent the SEM. The Sham conditions are averaged over Sham1 (before AG-cTBS) and Sham2 (after AG-cTBS), as there was no significant difference between those conditions.

Confirming our hypothesis regarding an early contribution of the left pSTG/STS, the analysis of the noun position revealed a significant interaction between Semantic expectancy × TMS condition. The classical N400 effect was smaller in the pSTG/STS condition (β = −1.040, *SE* = 0.399, *t* = −3.070, and *P*< 0.01) compared to the Sham2 condition of that particular session (β = −2.270, *SE* = 0.399, *t* = −6.691, and *P*< 0.001), indicating the causal relevance of the left pSTG/STS during the early time-window (Fig. 2).

Given that the reversed N400 window (400 to 600 ms) was not strongly contaminated by muscle-related artifacts resulting from the early rTMS perturbation (0 to 200 ms), we conducted an additional analysis on the verb position. Consistent with our observations at the noun position, a significant interaction of Semantic expectancy × TMS condition was revealed. Pairwise contrasts for each TMS condition indicated a significant reversed N400 effect in the Sham2 condition (β = 0.810, *SE* = 0.178, *t* = 4.546, and *P*< 0.001), but not in the pSTG/STS condition (*P*> 0.05). Consistent with the predictive coding account (63, 64), processing in the left pSTG/STS was thus (indirectly) important for the selectional restriction based on the verb, which, in turn, impacted the processing of the subsequent noun.

#### Effects of early pIFG-rTMS on the N400.

Regarding the analysis on the early contribution of the left pIFG, our findings indicated the presence of the classical N400 effect of the noun position (β = 0.906, *SE* = 0.117, *t* = 7.748, and *P*< 0.001) as well as the reversed N400 effect on the preceding verb position (β = −0.198, *SE* = 0.064, *t* = −3.840, and *P*< 0.01). In contrast to the left pSTG/STS, online rTMS over left pIFG during the early time-window did not modulate either of these effects (Fig. 2). This finding does not indicate a causal role of the left pIFG during this early rTMS window (in contrast to left pSTG/STS).

### Experiment 2.

The findings of Experiment 1 revealed the causal relevance of left pSTG/STS (but not left pIFG) for auditory sentence processing during the early rTMS time-window (0 to 200 ms). As a follow-up, Experiment 2 used a similar condition-and-perturb design (Fig. 1*C*) to address two specific research questions. First, Experiment 2 aimed at replicating the null findings following cTBS of left AG, as they seemed to contradict observations indicating its key role within the semantic network (47–49). Second, given that it is not a question of whether but rather when the left pIFG supports sentence comprehension (34), Experiment 2 further investigated the causal relevance of this brain region. To this end, the application of online rTMS over left pIFG was shifted to 150 ms after verb onset in Experiment 2 (Fig. 1*D*). The timing of rTMS (150 to 350 ms) was based on the available literature, which suggested that the activation propagation from the left temporal cortex to left inferior frontal brain regions might take about 200 to 400 ms after word onset (11, 12, 17, 56).

#### Replication of effects of AG-cTBS on the N400.

Our findings replicated the findings of Experiment 1 by showing that the mere application of left AG-cTBS did not have an impact on either processing window (all *P*> 0.05). Overall, however, the classical N400 effect was again larger at posterior electrodes compared to anterior electrodes (anterior: β = −1.71, *SE* = 0.347, *t* = −4.919, and *P*< 0.001; posterior: β = −2.26, *SE* = 0.347, *t* = −6.508, and *P*< 0.001). The reversed N400 effect at the verb position was also observed (β = −0.327, *SE* = 0.140, *t* = −2.342, and *P*< 0.05).

#### Effects of intermediate pIFG-rTMS on the N400.

Contrary to the findings of the early rTMS time-window (Experiment 1), modulation of the classical N400 effect was observed (β = −0.127, *SE* = 0.058, *t* = −2.201, and *P*< 0.05) when the online rTMS pulses over left pIFG were slightly delayed (150 to 350 ms) relative to verb onset (Fig. 2). More specifically, we observed a larger classical N400 effect in the Sham2 condition compared to the pIFG condition (Sham2: β = −1.80, *SE* = 0.331, *t* = −5.431, and *P*< 0.001; pIFG: β = −1.29, *SE* = 0.331, *t* = −3.901, and *P*< 0.001). Please note that the analysis on the reversed N400 effect is not reported here, as the verb position is too strongly contaminated by the longer-lasting stimulation-related artifacts (27–29) to be reliably interpreted.

### Experiment 3.

Thus far, the left posterior temporal cortex (0 to 200 ms) appears to be causally relevant prior to the left inferior frontal cortex (150 to 350 ms). This finding fits with earlier fMRI findings that also indicated a slightly earlier activation of the mid and posterior cortex compared to the IFG in adults (65). Crucially, however, Experiment 2 did not probe the functional contribution of the left pSTG/STS during the intermediate processing phase. To address this issue, Experiment 3 further tested the causal relevance of both left pIFG and pSTG/STS at the intermediate rTMS time point (150 to 350 ms), thereby also aiming to replicate the findings of Experiment 2 (pIFG) with an active control condition (vertex). In addition, Experiment 3 aimed to unravel whether the information flow between the temporal and frontal cortex is bidirectional during auditory sentence processing, as proposed by some recent models (3). To do so, we included a late rTMS time-window (300 to 500 ms). In order to answer both research questions in a single experiment, the design of Experiment 3 changed slightly in comparison with the previous two experiments (*Experimental Design*, and Fig. 1 *C* and *D*).

#### Effects of intermediate rTMS on the N400.

As a replication of Experiment 2, our findings again indicated the causal relevance of the left pIFG during the intermediate rTMS time-window, as reflected by a modulation of the classical N400 effect (β = −0.133, *SE* = 0.062, *t* = −2.161, and *P*< 0.05). More specifically, we observed the classical N400 effect in the vertex condition (β = −0.820, *SE* = 0.261, *t* = −3.137, and *P*< 0.01), but not in the left pIFG condition (*P*> 0.05; see Fig. 2). In contrast to the left pIFG and extending the findings of Experiment 2, the left pSTG/STS did not show significant causal relevance during this rTMS time-window.

#### Effects of late rTMS on the N400.

Interestingly, the opposite pattern as during the intermediate rTMS time point was observed when analyzing the causal relevance of our target regions during the late rTMS window (300 to 500 ms). Our findings showed that the left pSTG/STS was causally relevant during the late rTMS time point, as indicated by an interaction of Semantic expectancy × TMS condition (β = −0.158, *SE* = 0.065, *t* = −2.429, and *P*< 0.05). There was a classical N400 response in the vertex condition (β = −0.7232, *SE* = 0.184, *t* = −3.929, and *P*< 0.001), but not in the pSTG/STS condition (*P*> 0.05, see Fig. 2). The left pIFG, however, did not show significant causal relevance during this time point.

### Behavioral Results.

In the first two experiments, participants performed a lexical decision task on the sentence-final noun (i.e., classification as word or nonword). Therefore, we could investigate whether the observed impact of rTMS was also reflected on changes in behavioral performance. For each planned comparison, reaction times and response accuracy were analyzed with generalized linear mixed models (see *Behavioral Analysis* for more details). In brief, we observed the typical semantic expectancy effects on lexical decisions (36, 66–68), that is, faster and more accurate responses to highly compared to less expected nouns. This behavioral effect, however, was neither impacted by left AG-cTBS alone nor by the combined rTMS perturbation of two language nodes (all *P*> 0.05). We only observed general, nonspecific TMS effects on behavioral performance in the lexical decision task across both semantic expectancy conditions. Such nonspecific effects likely reflect facilitated behavior due to a general alerting effect after active brain stimulation (69). Full model summaries are presented in *SI Appendix*, Tables S4–S8.

The discrepancy between the behavioral and EEG results might be surprising at first given that both measures showed a strong influence of verb-based semantic expectancy. However, we are not the first to observe differential modulation of these measures see also ref. 70. For instance, the effects of constraint on the N400 response (68) show the opposite pattern as found for lexical decision times (71), indicating that contextual information can support processing at distinct times in different ways. From a causal perspective, our findings show that the lexical decision is not dependent on the processes reflected by the N400 response, as these measures were modulated independently from each other by rTMS.

The effect of rTMS on lexical decisions was not analyzed in Experiment 3, because the participants conducted a yes-no-probe detection task instead of a lexical decision task. On average, participants correctly detected the probe on 97.18% of trials (*SD* = 2.27%), indicating that they attended the sentence materials.

## General Discussion

**Fig. 3.**
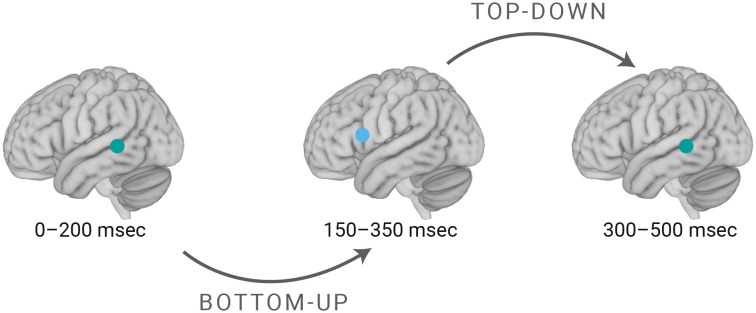
Overview of the results. Together, our findings provide evidence for the causal relevance of the left pSTG/STS in an early processing window (0 to 200 ms). The left pIFG was causally relevant in the intermediate processing window (150 to 350 ms), indicating a *Bottom-Up* (indirect) information transfer from the left pSTG/STS to the left pIFG. Finally, the left pSTG/STS again showed causal relevance in a late time-window (300 to 500 ms), suggesting *Top-Down* processing during auditory sentence comprehension.

Auditory sentence comprehension is known to be supported by a coordinated set of distributed and interconnected brain regions (3). By means of three condition-and-perturb TMS-EEG experiments, we provide insights that will extend current neurobiological models of language (3, 5, 6). We focused on three brain regions known to be involved in language comprehension: left pIFG, left pSTG/STS, and left AG (Fig. 1). We have provided much-needed causal and temporally specific evidence for the functional relevance of two key regions. The left posterior temporal cortex was involved early (0 to 200 ms), followed by an intermediate period (150 to 350 ms) where the left inferior frontal cortex became relevant, finally shifting back to the involvement of the left posterior temporal cortex (300 to 500 ms). An overview of the results of the three experiments is provided in Fig. 3. In our discussion below, we consider what the functional roles of these brain regions at the specific time-windows might be.

### Early Contribution of the Left Posterior Temporal Cortex.

During the early verb processing phase (0 to 200 ms), we observed that online rTMS perturbation of left pSTG/STS (indirectly) affected processing of the sentence-final noun. It is noteworthy that neither cTBS of left AG (Experiments 1 & 2) nor online rTMS perturbation of left pSTG/STS based on ref. 36 appears to impact processing by itself. Therefore, we suggest that the observed perturbation effects in the early time-window (0 to 200 ms) can be best explained by the summation effect induced by both rTMS protocols. Collectively, our present findings and previous work (36) indicate that the functional significance of the left pSTG/STS in the early time-window depends in part on the functional integrity of the left AG. In other words, the left AG, if not disrupted by cTBS, may help to maintain processing performance in the presence of a perturbed left pSTG/STS.

This finding is in agreement with lesion studies reporting comprehension deficits in patients with posterior temporal-parietal damage (50, 72–77). Given that these lesion studies cannot provide causal evidence at precise points in time (78), our TMS-EEG findings provide experimental evidence that, at least during the early rTMS window (0 to 200 ms), only the combined perturbation of both regions (AG-pSTG/STS) impacts processing (see *Limitations and Future Prospects*). There are several anchor points in the literature shedding light on what this interaction between left pSTG/STS and left AG might reflect. Despite the observation that stroke-induced brain lesions often tend to affect both regions (73), recent lesion-symptom studies suggest that the left pSTG/STS and left AG are functionally distinct (44). Most models (3, 5, 7, 10) posit a role for the left AG in lexical-semantic and/or conceptual-semantic processing, see also refs. 46–49, whereas the function of the left posterior temporal cortex is still discussed between them.

Several models have linked the left pSTG/STS to acoustic-phonological processing (3, 7, 10, 79). The early rTMS time-window (0 to 200 ms) indeed coincides with the N100, which reflects processes linked to the early acoustic-phonological analysis of speech (9). Moreover, converging neuroimaging evidence has linked the left pSTG/STS to phonological processes within this specific time window [∼100 ms; (54, 55, 80–82)]. Direct cortical recordings even specify that the auditory cortex comprising the transverse gyrus and the STG support phonological-level speech decoding (83–85). The STS appears to be functionally distinct, with the anterior and posterior dorsal bank of the STS being involved in lexical and phonological processing, respectively (86). Against this background, one could hypothesize that an uncertain acoustic-phonological analysis (due to pSTG/STS-rTMS) might result in weaker semantic constraints. Challenging listening conditions indeed seem to drive the neural system to build less expectancies, as the N400 amplitude has been shown to be reduced during degraded speech processing (87, 88).

Post hoc e-field calculations for the left pSTG/STS site indicated that the TMS-induced cortical stimulation also spread to the middle temporal gyrus (MTG; see Fig. 1*B* and *SI Appendix*, Fig. S1). This brain region is implicated in lexical processing based on current models of language comprehension (3, 6, 7, 10, 89), neuroimaging findings (47), and lesion-deficit studies (50, 90). Investigations of the time-course of lexical processing indeed indicate that access to words can already take place within our early time-window (91). Therefore, we suggest that the early rTMS application (0 to 200 ms) most probably impacted these bottom-up processes. Thus far, it appears that acoustic-phonological analysis is implemented by the auditory cortex, STG, and STS (posterior dorsal part), whereas STS (anterior dorsal part) possibly together with MTG support lexical processes.

The precise role of the left posterior MTG in lexical processing is subject to debate. Some researchers (7) have suggested that this brain region supports lexical-based syntactic processes. Lesion-deficit studies have reported evidence for this association (44, 75). Given the temporally imprecise nature of lesions, the observed syntactic comprehension deficits in these studies could likely also relate to the later (300 to 500 ms) causal contribution of the left posterior temporal cortex (discussed below). Other researchers suggest that this brain region rather plays a crucial role in lexical-semantic processing (3, 6, 10, 89), specifically in the retrieval of the word’s meaning from the lexicon. As such, rTMS (0 to 200 ms) might have impacted the mapping from phonological to lexical-semantic representations during sentence comprehension.

As described earlier, our findings of Experiment 1 in conjunction with ref. 36 point to an interaction between left AG and left pSTG/STS. Therefore, we suggest that the contribution of the left AG only becomes apparent as soon as processing is more challenging. This could be in terms of increased perturbation load, as in our experiments, or noisy listening environments, as previously observed (34, 41). In both of these challenging situations, high-level semantic knowledge from the left AG (47, 92) might be helpful for comprehension. One possible explanation for our findings is that during unifocal perturbation of left pSTG/STS in the early time-window (36), the left AG could still help to link the difficult-to-process input (due to rTMS) to higher-level semantic concepts. This functionality seems to become unnecessary during easier processing situations, thereby explaining why left AG-cTBS alone did not impact processing. This latter finding suggests that the involvement of the left AG in sentence comprehension probably depends on the respective processing demand see also refs. 78 and 93.

The interaction between the left AG and left pSTG/STS is likely mediated by anatomical fiber connections. The left AG is connected to posterior temporal regions via the (posterior) middle longitudinal fasciculus [MdLF, (52, 53)]. Consistent with our findings, the MdLF is suggested to be related to language processing (90, 94); but see ref. 95, particularly in the acoustic-phonetic processing of words (96). Our findings indirectly demonstrate that this connectivity is of functional significance for semantic aspects of auditory sentence comprehension. Similarly, white matter alterations of the MdLF in the dominant hemisphere have been associated with impairments in semantic processing and lexical retrieval in primary progressive aphasia (97).

### From Left Posterior Temporal to Prefrontal Cortex.

Following the early contribution of the left pSTG/STS, we found the causal relevance of the left pIFG emerging in a later time-window (150 to 350 ms). This observation extends earlier correlational MEG findings which also implied a sequential involvement of the left posterior temporal cortex followed by the left inferior frontal cortex (11–13, 16, 17, 56). Although our data clearly show a sequential causal involvement, the precise functional role of the left pIFG in sentence comprehension is still disputed.

The dominant view is that the left pIFG Brodmann Area [BA] 44 is involved in (morpho-)syntactic aspects of comprehension (3, 98, 99). This brain region is consistently activated for syntactic comprehension manipulations in several neuroimaging studies (9, 98, 100–103) Interestingly, the time-window (150 to 350 ms) in which we observed causal relevance of the left pIFG (BA44) overlaps with the time-window of two syntactic negativities (104): the early left anterior negativity (150 to 200 ms) indexing initial syntactic structure building and the left anterior negativity (300 to 400 ms) related to morpho-syntactic processing. Evidence from TMS during two-word constructions (e.g., “the piano”) showed that the disruption of the left pIFG during the second word affected syntactic processing, as indicated by a grammatical judgement task (105). Taking all these findings into account, left pIFG perturbation might have disrupted structure-based processing required for subsequent sentence-based conceptual-semantic processing. In line with this idea, ERP studies showed that earlier syntactic difficulties can block further semantic processing (106, 107). A recent temporally specific TMS-EEG study (27) shows that the left pIFG (BA44) is not causally involved in syntactic categorical predictions, but rather in syntactic composition.

In contrast to this dominant view, some researchers (1, 7) have argued against the left pIFG as a core syntactic comprehension region. Instead, this brain region has been linked to top-down selection and cognitive control (1, 7, 10). Within this view, the left pIFG has been suggested to exert top-down constraints on the upcoming argument(s) (108). Based on lesion evidence, it is difficult to determine whether the left pIFG has a top-down predictive or bottom-up detection role in syntactic comprehension, as by definition this brain region is impacted during both phases see refs. 27 and 99. Even though both views have their own merits, we prefer to interpret our findings in terms of the dominant view, at least in light of the currently available evidence.

### From Left Prefrontal Back to Posterior Temporal Cortex.

Some models (2, 89, 98) have argued that the left inferior frontal cortex is the dominant region where the unification of syntactic and semantic information takes place. In contrast, other models (3) proposed that semantic and syntactic integration rather takes place in the left posterior temporal cortex. The latter proposal sets the prerequisite that there is an information flow from the left inferior frontal cortex back to the left posterior temporal cortex. Supporting this prerequisite, recent MEG findings hinted toward a top-down influence of the IFG back to the pSTG at a later time-window (11, 16). Dorsal pathways connecting the left IFG and left pSTG/STS that could support such top-down processing have been proposed extensively (109).

Building further on these earlier findings, we found a causal role for the left pSTG/STS during a later verb rTMS window (300 to 500 ms) rather than the left pIFG (150 to 350 ms). Within the available literature, these brain regions have been suggested to form a fronto-temporal network responsible for thematic role assignment (18, 22, 99, 102). Beside an early role for the left posterior superior temporal cortex in basic acoustic processing (see above), neuroimaging (110–112) and lesion studies (113) indeed consistently reported that this brain region also comes into play when the assigned thematic roles need to be evaluated. Furthermore, effects of verb argument structure complexity have been observed in the left posterior temporal cortex (114–116). Based on these findings, we suggest that the role of the left pSTG/STS in the late time-window (300 to 500 ms) likely relates to thematic role assignment. These processes are important prerequisites for semantic preactivation of upcoming noun arguments based on the verb argument structure (i.e., reversed N400) to take place at the verb (35).

Alternatively, the left pIFG might have exerted top-down control over the left pSTG/STS during the processing of highly predictive verbs to place constraints on the upcoming noun (17). As such, it is tempting to suggest that left IFG, in particular its anterior part (BA45), is involved in the generation/maintenance of semantic predictions whereas the pSTG is associated with cortical representations of predicted elements and their integration into the context see also refs. 117, 118, and 108. Further supporting such top-down predictive coding, an MEG study (119) showed that STG neurons represent the difference between predicted and heard speech sound (i.e., prediction error). Similarly, another MEG study (16) showed activation of the pSTG evoked by unexpected compared to expected speech. Taking these findings into account, two brain regions appear to be involved in semantic processing during comprehension. While pIFG-rTMS (150 to 350 ms) might have impacted the top-down selectional prediction, pSTG/STS-rTMS (300 to 500 ms) could have perturbed the resulting cortical representations related to these selected elements.

### Limitations and Future Prospects.

Although there is no direct evidence for the discussed functional roles of our target regions (pIFG, pSTG/STS, and AG) in the present study, our suggestions are strongly motivated by the available neuroimaging, electrophysiological, brain stimulation, and aphasia literature. Clearly, future studies need to confirm these hypotheses.

Based on our recent TMS-EEG study (36), we know that unifocal perturbation of left pIFG or pSTG/STS (i.e., without preceding AG-cTBS) at verb onset was not sufficient to impact sentence-based semantic processing. It remains to be seen whether left AG-cTBS is also a prerequisite for the observed effects in the intermediate (pIFG) and/or late (pSTG/STS) time-window. We are confident, however, that the observed effects in the present study did not merely result from left AG-cTBS alone. Future research needs to confirm whether the observed effects in the intermediate (150 to 350 ms) and late time-windows (300 to 500 ms) resulted from unifocal or a combined perturbation. Studies only applying online rTMS over the left pIFG or pSTG/STS (without left AG-cTBS) in these time-windows could help to further address these open questions. Such studies would also offer deeper insights into the functional interactions within the language network that could form a robust system capable of withstanding unifocal perturbation.

Additionally, the observed modulation of ERPs following a combined perturbation of two nodes (Experiment 1) most likely originates from a modulation of the effective connectivity in the language network. Beyond local excitability changes in the left AG, the neural consequences of cTBS likely propagated throughout the whole functional (language) network (40, 120–122) and/or to domain-general regions (42). Even though the core language network has been well-defined (9), very little is known about the neural underpinnings of the flexible redistribution in the language network after left AG-cTBS (78). Consequently, it is unclear how AG-cTBS impacted the other nodes of the language network. A future combined TMS-fMRI study during sentence processing could help to reveal the impact of left AG-cTBS in the current experiments and to provide more information about flexible adaptation in the language system.

Notice that the triple-pulse rTMS protocols (10 Hz) used in the present experiments covered a 200 ms time-window. This larger time-window was necessary for an initial exploration of whether TMS was applied correctly in “time” given that there was insufficient information about when our target regions made a critical contribution (123). Our set of TMS-EEG experiments pave the way for future single-pulse TMS studies with an even more fine-grained tracking of the time course (chronometry) of the causal relevance of the left pSTG/STS and pIFG during language processing. One limitation of the current experimental design is the small (50 ms) overlap between the three time-windows (Fig. 1), which influences any direct comparisons of left pIFG versus left pSTG/STS in the present study due to residual perturbation effects (*SI Appendix*, Tables S22 and S23). Consequently, further experiments with nonoverlapping time-windows are required to provide stronger evidence for their distinct contributions at different time points during sentence comprehension.

Given that the present data do not allow to tease apart the contribution of the superior versus middle part of the posterior temporal cortex, future studies need to further address the question whether the early causal role of the left posterior temporal cortex (0 to 200 ms) is specifically related to phonological and/or lexical processing. Given the available literature, a tentative hypothesis would be a very early contribution of the left STG (∼100 ms) in phonological processing followed by a contribution of the left MTG (∼200 ms) in lexical processing.

## Conclusion

The present set of experiments highlight the coordinated spatiotemporal dynamics of the processing of words during auditory sentence comprehension. We show that this process is supported by the communication between the left posterior temporal and inferior frontal cortices, with a temporal dissociation with respect to the causal contribution of these brain regions. We observed a causal contribution of the left posterior temporal cortex during an early word processing time-window, followed by the relevance of the left inferior frontal cortex at an intermediate time-window. Finally, the left posterior temporal cortex showed again a contribution at the later time-window of word processing. Depending on the processing demands, the processes in these brain areas might be supported by the left inferior parietal cortex. To date, these concurrent TMS-EEG experiments are the first to provide causal, time-specific evidence for a coordinated temporal interplay of different brain regions during auditory sentence processing. As such, they provide an important extension of the insights gained by previous neuroimaging and lesion-deficit studies on the functional organization of the language network in the human brain.

## Materials and Methods

### Participants.

In each experiment, we report data from 24 (different) native German speakers (18 to 35 y; males and females) with normal or corrected-to-normal vision and no hearing deficits (*SI Appendix*, *Materials and Methods* and Table S1). All participants were right-handed according to the Edinburgh Inventory (124). Exclusion criteria were left-handedness, early bilingualism, a history of psychiatric or neurological disease, and any contraindications against TMS. Participants had a medical briefing for TMS safety prior to participation. The experiments were in concordance with the prerequisites of the guidelines of the Declaration of Helsinki and received approval from the Ethics committee of University of Leipzig (118/16-ek; 563/20-ek). All participants gave written informed consent prior to the study and received a monetary compensation for their participation.

### Experimental Design.

In Experiment 1, participants were invited for two sessions with an intersession interval of at least 6 d between the sessions (7.42${}_{M}$± 1.28${}_{\mathrm{SD}}$ d). In Experiment 2 to 3, participants were only invited for one session. Every experiment adopted a condition-and-perturb approach with three blocks per session that varied in the online rTMS target site (Fig. 1*C*). In Experiments 1 & 2, the first experimental block was always the ineffective online rTMS condition (Sham1), which was included to investigate the effect of offline cTBS of left AG on sentence-based semantic processing (i.e., cTBS effect: Sham1 vs. Sham2). The remaining two blocks were either the ineffective (Sham2) or effective online TMS condition (left pIFG or left pSTG/STS). In Experiment 1, effective online rTMS was applied over either left pIFG or pSTG/STS in separate sessions. The order of rTMS conditions applied after cTBS was completely counterbalanced across participants such that all combinations occurred equally often (Experiments 1 & 2).

The experimental design of Experiment 3 changed slightly compared to the first two experiments. Given the null finding following unifocal offline cTBS of left AG (Experiments 1 & 2), we no longer included a sham condition before the cTBS application (i.e., no Sham1). Consequently, cTBS was applied at the beginning of the experiment, followed by three blocks that differed in their online rTMS condition (pIFG, pSTG/STS, or vertex). Instead of an ineffective sham, online rTMS was applied over a control site (vertex) to confirm that any effects were specific to the stimulated areas (125). The order of rTMS conditions was counterbalanced.

### Tasks.

During the experimental blocks, participants listened to the German sentence materials (i.e., pronoun–verb–article–noun; see *Stimuli* for details) while EEG was recorded. Participants were instructed to fixate a fixation cross that was displayed throughout the blocks and to blink as little as possible. In Experiments 1 & 2, participants performed a lexical decision task on the sentence-final noun, indicating as fast and as accurate as possible whether it was a real word or a pseudoword via a button-press. Reaction times were measured starting at the onset of the critical noun/pseudoword. Responses exceeding 2,000 ms were counted as misses cf. ref. 36. In Experiment 3, participants performed a yes-no probe detection task following some trials (12.5%), indicating as fast and accurate as possible whether they heard the visually present word (a particular verb or noun) in the previous sentence.

### Stimuli.

Participants listened to German four-word sentences taken from our previous study (36). We included 80 experimental sentences that either had a low (< 25%; 15.3%${}_{M}$; see ref. 126) or high cloze probability (> 56%; 74.2%${}_{M}$) for their sentence-final noun. In Experiments 1 & 2, we presented the full set of items to each participant, whereas Experiment 3 only presented a randomly selected subset of 64 items per participant. More specifically, low cloze sentences (e.g., *He sees the beer*) were difficult to predict, because the verb could be linked to multiple nouns. In comparison, high cloze sentences (e.g., *He drinks the beer*) were easier to predict, because only a small number of nouns were likely to follow the highly predictive verb. In addition to the experimental items, filler items were included with a medium cloze probability in Experiments 1 & 2. Because participants performed a lexical decision task, half of the sentence materials (100 sentences) ended with pseudowords. To preserve novelty, a new set of pseudowords was used in each session. For more detailed information regarding the stimulus materials, see our previous study (36).

### Procedure.

Each experimental session started with EEG preparation and individual stereotaxic coregistration. The resting motor threshold (RMT) was determined in the first session (see *TMS Targets* for details). Before the start of the experimental blocks, participants practiced the task. During the practice session, sound volume was individually adjusted for each subject. Stimuli were presented via TMS-compatible in-ear headphones (SE215, Shure, Niles, IL).

During the main experiment, neuronavigated rTMS was applied over different target regions per experimental block (Fig. 1). Each block (± 10min) was divided into four subblocks with a short break every ± 2.5 to 3min. A randomized stimulus list was created for each participant and each block. The duration between sentences was jittered (range: 1,205 to 1,395 ms). Stimulus presentation and response recording was controlled via the Presentation® software (version 17.2, Neurobehavioral Systems, Inc., Berkeley, CA, www.neurobs.com).

### TMS Targets.

To account for anatomical individual variability, neuronavigated rTMS was performed using Montreal Neurological Institute (MNI) coordinates (pIFG: x, y, z = −60, 12, 16; pSTG/STS: x, y, z = −50, −42, 2; AG: x, y, z = −46, −64, 38) taken from a previous fMRI study (34) that were backtransformed to the individual MRI native space (Fig. 1*A*).

For the offline condition, the coil was navigated to the left AG and 40 s of cTBS (consisting of 50 Hz bursts of three pulses delivered at 5 Hz for a total of 600 stimuli; 43) were applied. This cTBS protocol was expected to temporarily reduce the excitability of the left AG. These after-effects often emerge with a certain delay (43) and wash out after around 30 to 60 min (127). Stimulation intensity for cTBS was 80% of the individual RMT, cf. refs. 121 and 128.

For online rTMS, the coil was navigated to left pIFG or left pSTG/STS. Stimulation intensity for online rTMS was 90% of individual RMT, cf. refs. 36 and 129. To compensate for the depth of our targets, its stimulation intensity was corrected following a simple linear correction (130). During each trial, an online TMS burst of three pulses with a frequency of 10 Hz was applied. Motivated by previous studies showing that contextual information is used in order to generate predictions about upcoming sentence-final nouns (35, 36), we applied online rTMS either at verb onset (Experiment 1), 150 ms after verb onset (Experiment 2 to 3), or 300 ms after verb onset (Experiment 3). The two timings (intermediate, late) used in Experiment 3 were pseudorandomly distributed within each block. The control rTMS condition consisted of either ineffective (Experiments 1 & 2) or effective rTMS over vertex (Experiment 3).

The rTMS pulses were controlled via the Presentation® software (version 17.2, Neurobehavioral Systems, Inc., Berkeley, CA, www.neurobs.com). TMS was applied using a figure-of-eight coil (C-B60; outer diameter 7.5 cm) connected to a MagPro X100 stimulator (MagVenture, Farum, Denmark). More details with respect to the TMS parameters can be found in *SI Appendix*, *Materials and Methods* and Table S2.

### Electric Field Simulations.

To assess the cortical stimulation exposure on the individual level, we calculated the induced e-fields using SimNIBS/charm [v4.0.1; for head-model construction and e-field simulation; (131, 132)] and pyNIBS [v0.2023.3; for subsequent analyses; (133)]. For each individual, one head mesh was constructed from high-resolution structural MRI scans (T1- and, if available, T2-weighted images). Two subjects were excluded due to meshing errors. For all subjects, individuals’ e-field were computed for the three target TMS conditions AG, pIFG, and pSTG/STS based on the coil placements and stimulator intensities used during the experimental sessions. One subject was excluded due to missing data.

### EEG.

The EEG was recorded from 63 Ag/AgCl electrodes [61 electrodes embedded in an elastic cap (EC80, EasyCap, GmbH, Herrsching, German), with additional electrodes on the left and right mastoids (A1, A2)], positioned according to the 10 to 20 system. Signals were recorded at a sampling rate of 2,000 Hz using a REFA8 68-channel amplifier system (TMSi, Oldenzaal, the Netherlands), grounded to the sternum, and referenced online to the average of all 63 electrodes. A linked mastoid reference was calculated offline. Electrode impedances were kept below 5 kΩ. To minimize TMS-induced artifacts, the electrode leads were placed orthogonal to the current flow in the coil cf. ref. 134. Data acquisition was done using the Brain Vision Recorder software (Brain Vision, MedCaT B.V.). The electrooculogram (EOG) was measured from the outer sides of both eyes and from the top and bottom of the right eye. The EEG signal was monitored throughout the experiment. Electrode positions were digitized and coregistered to the individual anatomical MRI using a frameless stereotaxic neuronavigation system (TMS Navigator, Localite, GmbH, Sankt Augustin, Germany).

### EEG Data Processing.

Due to the on-going developments of new artifact-rejection techniques within the cutting-edge TMS-EEG field (135, 136), the preprocessing of EEG data was different in Experiment 1 and 2 compared to Experiment 3. At the time that the first two experiments were conducted, using two rounds of independent component analysis [ICA; (137)] was the state-of-the-art TMS-artifact rejection technique (*SI Appendix*, Table S3). By the time Experiment 3 was conducted, however, large advances in source-based artifact-rejection techniques for TMS-EEG (138) were made, where strong assumptions regarding the statistical independence of the noise and artifact signals are no longer needed.

#### Experiment 1 and 2.

EEG data were preprocessed offline using the FieldTrip toolbox (139) in Matlab (The Mathworks, USA). The large magnetic pulses were removed and interpolated (−2 to 20 ms) using a cubic function. Two rounds of ICA (137) were performed to remove large rTMS-related artifacts (round 1) and other nonneural artifacts (round 2). The continuous EEG data were both high-pass [0.1 Hz, (140)] and low-pass filtered [30 Hz; kaiser-windowed sinc-FIR-filter; deviation: 0.001, (36)]. Next, the EEG data were rereferenced to an average of A1 and A2 (linked mastoid). Our main window-of-interest was time-locked to the onset of the sentence-final noun (−200 to 1,000 ms). For some additional control analyses, we also report the ERPs time-locked to the onset of the midsentence verb (−250 to 1,000 ms). For both positions, trials exceeding a range of 150 µV cf. ref. 36 were automatically rejected. A 200-ms prestimulus baseline was applied for both the noun (−200 to 0 ms) and verb position [−250 to −50 ms, (36)]. A 10-Hz low-pass filter was used for visualization purposes only.

#### Experiment 3.

To enhance the reproducibility of TMS-EEG studies (141), we fully automated our preprocessing pipeline for Experiment 3. Offline EEG preprocessing was performed using a combination of EEGLAB (142), the TMS-EEG signal analyzer (TESA) toolbox (143), and FieldTrip (139) in the Matlab environment (The Mathworks, USA). The raw EEG signal was epoched relative to verb onset (−500 to 2,500 ms), bad channels were automatically detected (via EEGLAB’s *pop_rej_chan* function run twice; rejected if > 3.5${}_{\mathrm{SD}}$ or <−5${}_{\mathrm{SD}}$ from mean power), and the large TMS artefact (−2 to 20 ms) was interpolated using a cubic function. ICA was applied to the segmented data and ICLabel (144) was used to automatically identify and exclude ICs that had a high probability (>80% eye and <10% brain) of being classified as reflecting eye-related artifacts. Then, SOUND [source-estimate-utilizing noise-discarding algorithm; lambda = 0.01, 5 iterations, (138)] and SSP-SIR [signal-space-projection-source-informed reconstruction; fixed criterion: 90% variance, (145)] with subject-specific lead-field models [boundary element method, (145)] were used to further clean the signal. The conductivities of brain, skull, and skin set to 0.33 S/m, 0.033 S/m, and 0.33 S/m, respectively (145). Moreover, EEG data were low-pass (30 Hz) and high-pass (0.1 Hz) filtered using a Hamming windowed sinc FIR filter (via EEGLAB’s *pop_eegfiltnew* function), and then rereferenced to a linked mastoid. Bad trials were automatically detected and removed based on the joint probability of the data (via EEGLAB’s *pop_jointprob* function applied to channels-of-interest, *SD*= 5). We used a 100 ms prestimulus baseline (−100 to 0 ms) for the noun analysis cf. ref. 37 to avoid any impact of the TMS-pulses.

### Behavioral Statistical Analysis.

For each planned comparison, statistical analyses on reaction times and response accuracy were computed with generalized mixed-effects models fit by maximum likelihood (Laplace Approximation) using the function glmer from the lme4 package (146) in R (147) . Contrary to linear mixed effects models, generalized mixed effects models can account for the right-skewed shape of the reaction time distribution without having to transform the raw data (148). For the reaction time data, we fitted an identity function assuming a Gamma distribution (i.e., right skewed with a long tail in the slow reaction times). Response accuracy (0 = incorrect, 1 = correct) was analyzed using mixed logit regression (149). The factors Semantic expectancy (low cloze vs. high cloze) and TMS condition (Sham1 vs. Sham2; Sham2 vs. pSTG/STS; Sham2 vs. pIFG) were sum-coded (−1 vs. 1) and included as fixed effects. Using the buildmer package (150, 151), a maximal random effects structure with random intercepts and slopes for participant and item (for all fixed effects and their interaction) was included as long as the model would still converge (152); but see ref. 153. For the comparison of both sham conditions (i.e., cTBS effect: Sham1 vs. Sham2) in Experiment 1, random intercepts and slopes were additionally included for Session (first vs. second). In brief, the buildmer function entered the random effect terms in order of their contribution according to likelihood-ratio tests, such that when the model eventually failed to converge, the most information-rich random effects were included. Reported *P* values were based on asymptotic Wald tests implemented in the lme4 package. The alpha level was set to < 0.05 for all analyses.

### EEG Statistical Analysis.

Similar to the behavioral analyses, we conducted several planned comparisons to address our research questions. For each experiment, we report the results of a series of linear mixed-effect regression models fit by restricted maximum likelihood using the function lmer from the lme4 package (146) in R (147). Our dependent measure was the trial-level ERP amplitude averaged across electrodes within four ROIs (36): anterior left (AF3, F5, F3, FC5, FC3, and FC1), anterior right (AF4, F6, F4, FC6, FC4, and FC2), posterior left (CP5, CP3, CP1, P5, P3, and PO3), and posterior right (CP6, CP4, CP2, P6, P4, and PO4) during a particular time-window of interest for the noun [300 to 500 ms, (33, 36)] and verb position [400 to 600 ms, (36)].

For each analysis, the factors Semantic Expectancy (low cloze vs. high cloze), TMS condition (control vs. effective target), Anteriority (anterior vs. posterior) and Laterality (*Left* vs. *Right*) were sum-coded (−1 vs. 1) and included as fixed effects. Again, we used buildmer to automatically determine the maximally feasible model. As a starting point, the maximal random effects structure was used (152) with by-item and by-subject random intercepts and by-item and by-subject random slopes for each fixed effect of interest and their interaction. Random intercepts and slopes were additionally included for Session for the comparison of both sham conditions (i.e., cTBS effect: Sham1 vs. Sham2) in Experiment 1. For each contrast of interest, *P* values were estimated using a Satterthwaite approximation, as implemented by the lmerTest package (154), see ref. 155. We used the package emmeans (156) for pairwise follow-up comparisons to further explore significant interactions. *P* values below an alpha level of 0.05 were considered significant.

## Supplementary Material

Appendix 01 (PDF)

## Data Availability

Preprocessing scripts and statistical analysis code can be found at the OSF (https://osf.io/tb58w/?view_only=6850973440b94fedadc827e37a507e89) (157). The conditions of our ethics approval and consent procedures do not permit public archiving of anonymized study data. Therefore, this data will be made available upon request.
