# Supplementary material for: Causal evidence for a coordinated temporal interplay within the language network
Source: Proc Natl Acad Sci U S A. 2023 Nov 14;120(47):e2306279120. doi: 10.1073/pnas.2306279120 (PMC10666120; doi:10.1073/pnas.2306279120)
Supplement: Supplementary file 1 — Appendix 01 (PDF) [file pnas.2306279120.sapp.pdf]

Supporting Information for

Causal Evidence for a Coordinated Temporal Interplay within the Language Network

Joëlle A. M. Schroën, Thomas C. Gunter, Ole Numssen, Leon O. H. Kroczek, Gesa Hartwigsen, Angela D. Friederici

Corresponding Author: Joëlle A. M. Schroën
E-mail: schroen@cbs.mpg.de

This PDF file includes:

Supporting text
Figs. S1 to S2
Tables S1 to S23
SI References

Supporting Information Text

SI Results

Behavioral Results.

Experiment 1: Early rTMS perturbation (0-200 msec).

Effects of AG-cTBS on behavioral performance Analysis of response accuracy indicated a non-specific main effect of *TMS condition* with overall higher accuracy before cTBS (Sham1: 97.4% correct) compared to after cTBS (Sham2: 96.5% correct; β = -0.244, SE = 0.118, z = -2.071, p < 0.05). Reaction times yielded a main effect of *Semantic expectancy* (β = -23.338, SE = 1.638, t = -14.245, p < 0.001), showing that responses for high cloze sentences were faster (686 msec) than low cloze sentences (730 msec). In the discussed analyses, there were no significant interactions of Semantic expectancy x TMS condition, indicating that the observed behavioral differences between high and low cloze sentences remained unaffected by cTBS over the left AG (see Table S4).

Effects of pSTG/STS-rTMS on behavioral performance Analysis of response accuracy yielded a main effect of *Semantic expectancy* (Low cloze: 95.9% vs. high cloze: 98.2%; β = 0.610, SE = 0.124, z = 4.935, p < 0.001). Reaction times also showed a main effect of *Semantic expectancy* (Low cloze: 715 msec vs. high cloze: 670 msec; β = -22.328, SE = 2.221, t = -10.054, p < 0.001). No significant interactions of Semantic Expectancy x TMS condition were revealed. These findings indicate that the additional perturbation of left pSTG/STS (after offline conditioning of left AG) did not impact behavioral performance in the lexical decision task (see Table S5).

Effects of pIFG-rTMS on behavioral performance Response accuracy showed a main effect of *Semantic expectancy* (Low cloze: 96% vs. high cloze: 98%; β = 0.555, SE = 0.146, z = 3.817, p < 0.001). Analysis of reaction times also yielded a main effect of *Semantic expectancy* (Low cloze: 723 msec vs. high cloze: 687 msec β = -20.764, SE = 2.455, t = -8.459, p < 0.001). There was also a non-specific main effect of *TMS condition* (Sham2: 716 ms vs. pIFG: 696 ms; β = -6.855 SE = 2.468, t = -2.778, p < 0.01). Again, no significant interactions of Semantic expectancy x TMS condition were demonstrated, showing that additional perturbation of left pIFG (after offline conditioning of left AG) did not impact the observed behavioral differences between high and low cloze sentences in the lexical decision task (see Table S6).

Experiment 2: Middle rTMS perturbation (150-350 msec).

Effects of AG-cTBS on behavioral performance Response accuracy yielded no significant effects. Analysis of reaction times showed a main effect of *Semantic expectancy* (β = -21.583, SE = 3.629, z = -5.948, p < 0.001). Furthermore, a non-specific main effect of *TMS condition* was revealed (β = -14.539, SE = 2.479, t = -5.865, p < 0.001). Crucially, no interactions of Semantic expectancy x TMS condition were observed. Replicating our findings of Experiment 1, these findings indicate that cTBS over left AG did not impact the behavioral differences between high and low cloze sentences in the lexical decision task (see Table S7).

Effects of pIFG-rTMS on behavioral performance Analysis of response accuracy revealed no significant effects. Reaction times revealed a main effect of *Semantic expectancy* (β = -23.461, SE = 2.534, z = -9.257, p < 0.001). Furthermore, there was also a main effect of *TMS condition* (β = 6.700, SE = 2.462, t = 2.722, p < 0.01). No interaction of Semantic expectancy x TMS condition was revealed, indicating that this rTMS effect was non-specific (see Table S8).

SI Methods and Materials

Transcranial magnetic stimulation. Stereotaxic neuronavigation (TMS Navigator, Localite, GmbH, Sankt Augustin, Germany) was used to navigate the coil to the target areas and maintain its exact location and orientation throughout the experimental sessions. The participant's head was co-registered onto their individual T1-weighted MRI image at the beginning of each session (MPRAGE sequence in sagittal orientation, voxel size = 1 x 1 x 1.5 mm; TR = 1.3 s, TE = 3.36 ms; whole brain). The average Montreal Neurological Institute (MNI) coordinates for the left pIFG (x, y, z = -60, 12, 16; Brodmann Area [BA] 44), left pSTG/STS (x, y, z = -50, -42, 2), and left AG (x, y, z = -46, -64, 38; BA39) were taken from a previous fMRI study using similar Item materials (1). To target these coordinates individually, they were transformed from Montreal Neurological Institute (MNI) to Participant space using the SPM12 software (Welcome Trust Center for Neuroimaging, University College London, UK).

Stimulation intensity was corrected for the difference in scalp-cortex distance between the left primary motor cortex (M1) and the targets. The average MNI coordinates for the M1 were taken from a meta-analysis (x, y, z= –37, –21, 58 mm; 2). These coordinates were also used as a starting point for determining the individual resting motor threshold (RMT). The RMT was defined as the lowest stimulation intensity producing at least 5 visible motor evoked potentials of approximately 50 µV (peak-to-peak amplitude) in the relaxed first interosseus muscle of the right hand when single-pulse TMS was applied over left M1 (with EEG cap on). In Experiment 1, the individual RMT was held constant across sessions (cf. 3, 4).

For the distance correction, we followed the simple linear correction approach recommended by Stokes et al. (5). The distance correction was always applied to 90% RMT (instead of 100% RMT) to avoid unpleasantly high stimulation intensities (cf. 4, 6). If the individual stimulation intensity was too uncomfortable for the participant, it was gradually lowered to the highest intensity that was still comfortable. The intensity for left pIFG and left AG stimulation were not adjusted for distance

as their targets lay very close to the scalp (cf. 4). The corrected mean-stimulation intensity per active TMS target can be found in Table S2. In Experiment 1-2, the stimulation intensity of the ineffective sham conditions was always the same as the effective online rTMS condition (either pIFG or pSTG/STS) of that particular session.

TMS was applied using a figure-of-eight coil (C-B60; outer diameter 7.5 cm) connected to a MagPro X100 stimulator (MagVenture, Farum, Denmark). For the left AG, the coil was positioned with the handle pointing parallel to the sagittal plane (cf. 7). For the left pIFG, we oriented the coil 45° to the sagittal plane, with the second phase of the biphasic pulse inducing a posterior-to-anterior current flow (cf. 3, 4, 8, 9). Due to anatomical restrictions, coil placement of left pSTG/STS required rotation of the coil at an angle of 225° (cf. 3, 6). Consequently, the current flow was inversed. For sham rTMS in Experiment 1-2, an ineffective coil was placed over the vertex (i.e., electrode Cz) and an additional coil was placed over the first coil at a 90° angle (cf. 3, 9, 10). Only the second coil was charged, which created similar acoustic sensations compared to the effective online conditions without actively stimulating the brain. In Experiment 3, we used effective rTMS over vertex (Cz) instead of ineffective sham as a control condition. Participants were told that any differences in sensations between rTMS conditions were due to different locations of the coil on the head (cf. 11).

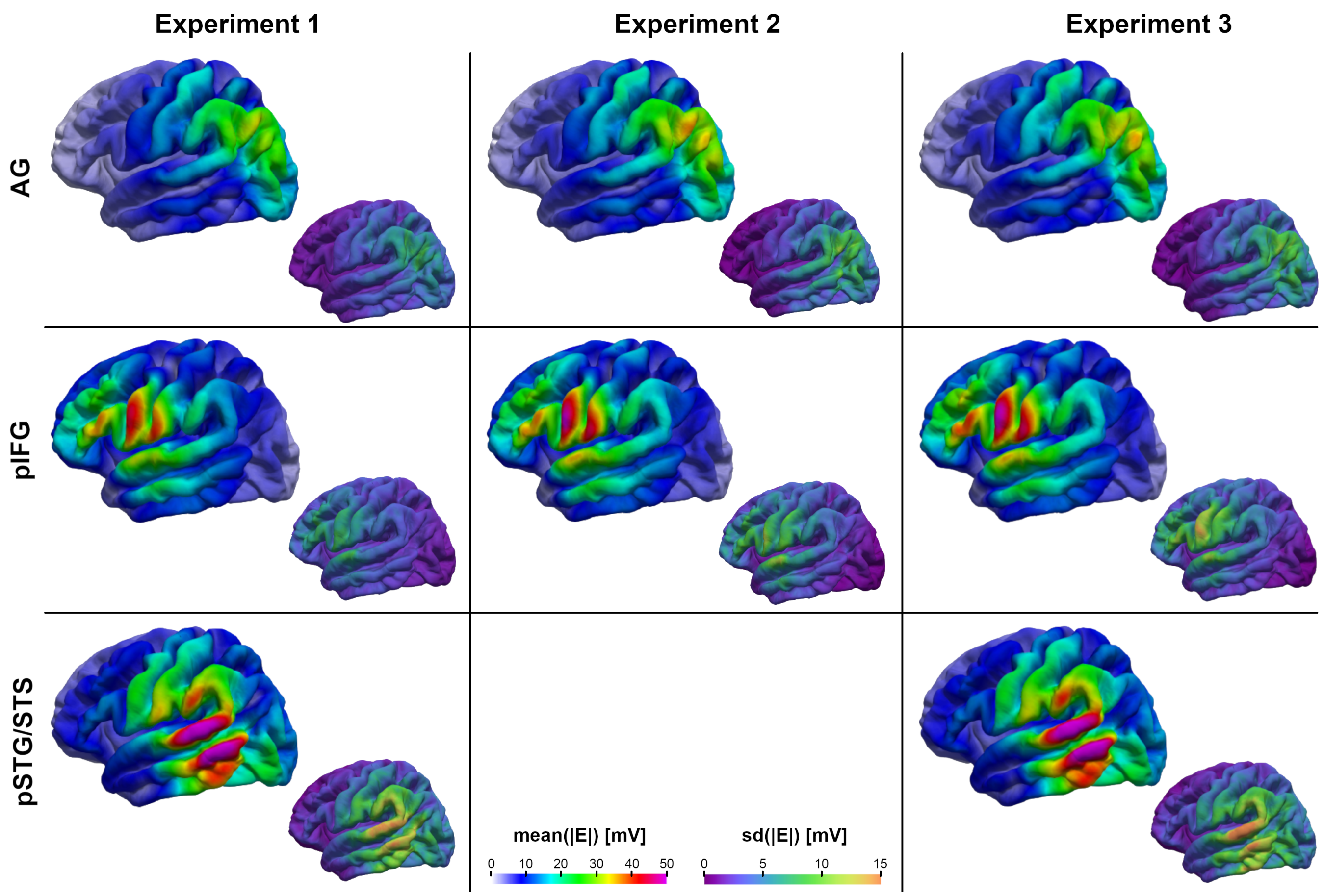

Fig. S1. Group averaged cortical field exposure for all three experiments. The induced electric fields (e-fields) for all experiments and target sites were computed individually and transformed into fsaverage template space. The offline TMS target, i.e. the angular gyrus (AG, upper row), was stimulated with 80% of the individual resting motor threshold (RMT). The online TMS targets, i.e. posterior inferior frontal gyrus (pIFG, middle row) and the posterior superior temporal gyrus/sulcus (pSTG/STS, bottom row), were stimulated with 90% RMT (plus Stokes distance correction, if applicable. Mean intensities per target can be found in Table S2. Large brain plots: average field exposure across subjects. Small brain plots: standard deviation of the e-field exposure across subjects.

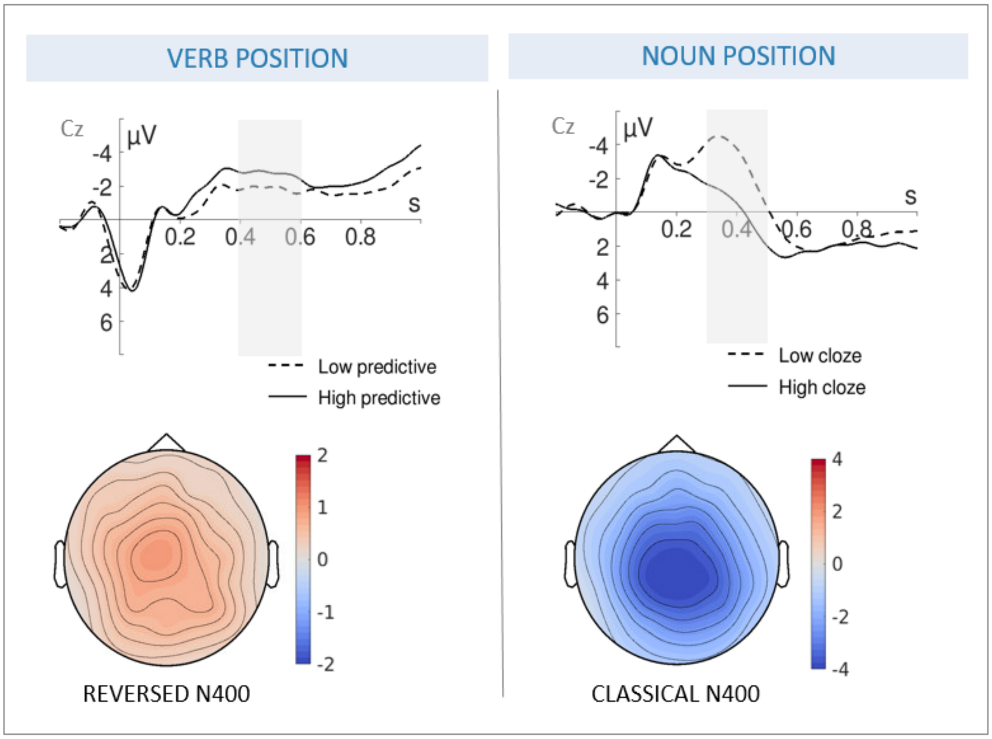

Fig. S2. Overview of the reversed N400 effect at the mid-sentence verb in Experiment 1. Mean reversed N400 amplitude for the sham condition, with the N400 operationalised as the average voltage across all time points between 400 and 600 msec across all electrode sites within our ROI. Negative voltage is plotted upward. The Sham condition is an average over Sham1 (before cTBS) and Sham2 (after cTBS), as there was no significant difference between those conditions.

Table S1. Participant Information per Experiment

Experiment	$Age_{(M \pm SD)}$	Laterality $Index_{(M \pm SD)}$	$Females_{(N)}$
Experiment 1 (N = 24)	28.17 ± 3.62	91.88 ± 9.95	13
Experiment 2 (N = 24)	27.12 ± 4.55	89.33 ± 11.05	12
Experiment 3 (N = 24)	27.71 ± 4.96	89.46 ± 10.66	13

Table S2. Stimulation Intensities per active TMS Target

Experiment	$pIFG_{(M \pm SD)}$	$pSTG/STS_{(M \pm SD)}$	$AG_{(M \pm SD)}$	$vertex_{(M \pm SD)}$
Experiment 1	45.92% ± 9.02%	61.75% ± 9.28%	42.46% ± 7.60%	N.A.
Experiment 2	50.25% ± 5.96%	N.A.	45.75% ± 4.59%	N.A.
Experiment 3	51.21% ± 6.70%	65.92% ± 8%	45.45% ± 6.05%	51.42% ± 6.93%

Table S3. Average number of removed ICs per dataset

Experiment 1

ICA Round	Sham1 (1)$_{(M \pm SD)}$	Sham2 (1)$_{(M \pm SD)}$	Sham1 (2)$_{(M \pm SD)}$	Sham2 (2) $_{(M \pm SD)}$	pIFG $_{(M \pm SD)}$	pSTG/STS$_{(M \pm SD)}$
Round 1	N.A.	N.A.	N.A.	N.A.	4.29 ± 1.76	6.25 ± 2.09
Round 2	23.88 ± 5.42	24.83 ± 5.27	28.58 ± 6.49	23.21 ± 5.32	20.63 ± 6.84	22.13 ± 5.53

Experiment 2

ICA Round	Sham1$_{(M \pm SD)}$	Sham2$_{(M \pm SD)}$			pIFG $_{(M \pm SD)}$	
Round 1	N.A.	N.A.			9.75 ± 2.45	
Round 2	19.58 ± 7.53	14.45 ± 6			9.92 ± 3.84	

Notes. Sham1 (1): Sham before cTBS in the first session; Sham1 (2): Sham before cTBS in the second session; Sham2 (1): Sham after cTBS in the first session; Sham2 (2): Sham after cTBS in the second session. Please note that a different preprocessing pipeline was used for Experiment 3.

Table S4. Experiment 1: Effect of AG-cTBS on behavioral performance

Response accuracy (ACC)

Model formula: ACC $\sim$ 1 + SEM + TMS + SEM:TMS + (1 + TMS + SEM | Item) + (1 | Participant)
Family: binomial (logit)

Fixed Effects	Estimate	SE	z	p	
(Intercept)	4.402	0.194	22.724	0.000	***
SEM1	0.208	0.153	1.363	0.173	
TMS1	-0.244	0.118	-2.071	0.038	*
SEM1:TMS1	0.017	0.111	0.153	0.879	

Reaction time (RT)

Model formula: RT $\sim$ 1 + SEM + TMS + SEM:TMS + (1 | Participant) + (1 | Item)
Family: Gamma (identity)

Fixed Effects	Estimate	SE	t	p	
(Intercept)	730.698	4.926	148.331	0.000	***
SEM1	-23.338	1.638	-14.245	0.000	***
TMS1	-2.166	1.673	-1.295	0.195	
SEM1:TMS1	0.821	1.669	0.492	0.623	

Sum-coding: TMS (-1 = Sham1, 1 = Sham2); SEM (-1 = low cloze, 1 = high cloze).

Significance codes: *** $<$ 0.001, ** $<$ 0.01, * $<$ 0.05, . $<$ 0.1

Table S5. Experiment 1: Effect of early pSTG/STS-rTMS on behavioral performance

Response accuracy (ACC)

Model formula: ACC ∼ 1 + SEM + TMS + SEM:TMS + (1 | Item) + (1 + TMS | Participant)
Family: binomial (logit)

Fixed Effects	Estimate	SE	z	p	
(Intercept)	4.420	0.261	16.925	0.000	***
SEM1	0.610	0.124	4.935	0.000	***
TMS1	0.075	0.146	0.510	0.610	
SEM1:TMS1	-0.012	0.123	-0.101	0.920	

Reaction time (RT)

Model formula: RT ∼ 1 + SEM + TMS + SEM:TMS + (1 | Participant) + (1 + TMS | Item)
Family: Gamma (identity)

Fixed Effects	Estimate	SE	t	p	
(Intercept)	717.141	5.975	120.029	0.000	***
SEM1	-22.328	2.221	-10.054	0.000	***
TMS1	-3.795	2.951	-1.286	0.198	
SEM1:TMS1	1.471	2.321	0.634	0.526	

Sum-coding: TMS (-1 = Sham2, 1 = pSTG/STS); SEM (-1 = low cloze, 1 = high cloze).

Significance codes: *** < 0.001, ** < 0.01, * < 0.05, . < 0.1

Table S6. Experiment 1: Effect of early pIFG-rTMS on behavioral performance

Response accuracy (ACC)

Model formula: ACC $\sim$ 1 + SEM + TMS + SEM:TMS + (1 | Item) + (1 + SEM | Participant)<br>Family: binomial (logit)					
Fixed Effects	Estimate	SE	z	p	
(Intercept)	4.562	0.283	16.118	0.000	***
SEM1	0.555	0.146	3.817	0.000	***
TMS1	0.149	0.123	1.217	0.224	
SEM1:TMS1	0.060	0.123	0.490	0.624	

Reaction time (RT)

Model formula: RT $\sim$ 1 + SEM + TMS + SEM:TMS + (1 | Participant) + (1 | Item)<br>Family: Gamma (identity)					
Fixed Effects	Estimate	SE	t	p	
(Intercept)	729.745	9.312	78.364	0.000	***
SEM1	-20.764	2.455	-8.459	0.000	***
TMS1	-6.855	2.468	-2.778	0.005	**
SEM1:TMS1	1.413	2.502	0.565	0.572	

Sum-coding: TMS (-1 = Sham2, 1 = pIFG); SEM (-1 = low cloze, 1 = high cloze).

Significance codes: *** < 0.001, ** < 0.01, * < 0.05, . < 0.1

Table S7. Experiment 2: Effect of AG-cTBS on behavioral performance

Response accuracy (ACC)

Model formula: ACC $\sim$ 1 + SEM + TMS + SEM:TMS + (1 + SEM | Item) + (1 | Participant)
Family: binomial (logit)

Fixed Effects	Estimate	SE	z	p	
(Intercept)	4.378	0.305	14.371	0.000	***
SEM1	0.110	0.172	0.638	0.523	
TMS1	0.022	0.104	0.211	0.833	
SEM1:TMS1	0.005	0.104	0.044	0.965	

Reaction time (RT)

Model formula: RT $\sim$ 1 + SEM + TMS + SEM:TMS + (1 + SEM | Participant) + (1 | Item)
Family: Gamma (identity)

Fixed Effects	Estimate	SE	t	p	
(Intercept)	832.593	8.437	98.684	0.000	***
SEM1	-21.583	3.629	-5.948	0.000	***
TMS1	-14.539	2.479	-5.865	0.000	***
SEM1:TMS1	1.571	2.596	0.605	0.545	

Sum-coding: TMS (-1 = Sham1, 1 = Sham2); SEM (-1 = low cloze, 1 = high cloze).

Significance codes: *** < 0.001, ** < 0.01, * < 0.05, . < 0.1

Table S8. Experiment 2: Effect of midtime pIFG-rTMS on behavioral performance

Response accuracy (ACC)

Model formula: ACC ∼ 1 + SEM + TMS + SEM:TMS + (1 + SEM | Item) + (1 | Participant)<br>Family: binomial (logit)					
Fixed Effects	Estimate	SE	z	p	
(Intercept)	4.491	0.313	14.332	0.000	***
SEM1	0.136	0.208	0.653	0.514	
TMS1	0.015	0.107	0.143	0.886	
SEM1:TMS1	0.042	0.107	0.394	0.694	

Reaction time (RT)

Model formula: RT ∼ 1 + SEM + TMS + SEM:TMS + (1 | Participant) + (1 | Item)<br>Family: Gamma (identity)					
Fixed Effects	Estimate	SE	t	p	
(Intercept)	821.930	6.924	118.712	0.000	***
SEM1	-23.461	2.534	-9.257	0.000	***
TMS1	6.700	2.462	2.722	0.006	**
SEM1:TMS1	-3.615	2.568	-1.408	0.159	

Sum-coding: TMS (-1 = Sham2, 1 = pIFG); SEM (-1 = low cloze, 1 = high cloze).

Significance codes: *** < 0.001, ** < 0.01, * < 0.05, . < 0.1

Table S9. Experiment 1: the effect of AG cTBS on the N400 at noun position

Linear Mixed-Effects Model (REML)

Fixed Effects	Estimate	SE	t	p	
(Intercept)	-1.111	0.316	-3.510	0.001	**
SEM1	0.991	0.118	8.370	0.000	***
anteriority1	0.972	0.042	23.098	0.000	***
laterality1	-0.370	0.042	-8.784	0.000	***
TMS1	0.129	0.042	3.071	0.002	**
anteriority1:laterality1	-0.137	0.042	-3.265	0.001	**
SEM1:anteriority1	0.134	0.042	3.186	0.001	**
anteriority1:TMS1	0.087	0.042	2.069	0.039	*
laterality1:TMS1	-0.038	0.042	-0.898	0.369	
SEM1:laterality1	0.034	0.042	0.808	0.419	
SEM1:TMS1	0.008	0.042	0.200	0.841	
SEM1:anteriority1:laterality1	0.029	0.042	0.679	0.497	
SEM1:anteriority1:TMS1	-0.018	0.042	-0.436	0.663	
SEM1:laterality1:TMS1	0.016	0.042	0.371	0.711	
anteriority1:laterality1:TMS1	-0.004	0.042	-0.094	0.925	
SEM1:anteriority1:laterality1:TMS1	0.004	0.042	0.094	0.925	

Pairwise Contrasts

Anteriority (Contrast	Estimate	SE	t	p	
Anterior (low - high)	-1.71	0.251	-6.821	0.000	***
Posterior (low - high)	-2.25	0.251	-8.954	0.000	***

Model formula: MEAN $\sim$ 1 + SEM + anteriority + laterality + anteriority:laterality + SEM:anteriority + TMS + anteriority:TMS + laterality:TMS + SEM:laterality + SEM:anteriority:laterality + SEM:TMS + SEM:anteriority:TMS + SEM:laterality:TMS + anteriority:laterality:TMS + SEM:anteriority:laterality:TMS + (1 | Participant) + (1 + SEM | Item)

Sum-coding: TMS (-1 = Sham1, 1 = Sham2); SEM (-1 = low cloze, 1 = high cloze).

Significance codes: *** < 0.001, ** < 0.01, * < 0.05, . < 0.1

Table S10. Experiment 1: The effect of AG cTBS on the N400 at the verb position

Linear Mixed-Effects Model (REML)

Fixed Effects	Estimate	SE	t	p	
(Intercept)	-1.367	0.233	-5.866	0.000	***
SEM1	-0.270	0.045	-5.960	0.000	***
anteriority1	-0.239	0.140	-1.713	0.100	
TMS1	-0.090	0.105	-0.852	0.403	
laterality1	-0.025	0.045	-0.563	0.573	
anteriority1:TMS1	0.096	0.045	2.118	0.034	*
SEM1:TMS1	-0.082	0.045	-1.812	0.070	.
SEM1:anteriority1	-0.028	0.045	-0.621	0.535	
TMS1:laterality1	0.022	0.045	0.488	0.625	
anteriority1:laterality1	-0.018	0.045	-0.390	0.696	
SEM1:laterality1	-0.007	0.045	-0.160	0.873	
SEM1:anteriority1:TMS1	-0.028	0.045	-0.627	0.531	
anteriority1:TMS1:laterality1	-0.024	0.045	-0.537	0.591	
SEM1:anteriority1:laterality1	-0.022	0.045	-0.487	0.626	
SEM1:TMS1:laterality1	0.017	0.045	0.384	0.701	
SEM1:anteriority1:TMS1:laterality1	0.000	0.045	0.005	0.996	

Model formula: MEAN $\sim$ 1 + SEM + anteriority + TMS + anteriority:TMS + SEM:TMS + SEM:anteriority + SEM:anteriority:TMS + laterality + TMS:laterality + anteriority:laterality + anteriority:TMS:laterality + SEM:laterality + SEM:anteriority:laterality + SEM:TMS:laterality + SEM:anteriority:TMS:laterality + (1 + anteriority + TMS | Participant) + (1 | Item)

Sum-coding: TMS (-1 = Sham1, 1 = Sham2); SEM (-1 = low cloze, 1 = high cloze).

Significance codes: *** < 0.001, ** < 0.01, * < 0.05, . < 0.1

Table S11. Experiment 1: The effect of early pSTG/STS-rTMS on the N400 at the noun position

Linear Mixed-Effects Model (REML)

Fixed Effects	Estimate	SE	t	p	
(Intercept)	-1.041	0.342	-3.040	0.005	**
anteriority1	0.864	0.058	14.981	0.000	***
SEM1	0.828	0.160	5.191	0.000	***
laterality1	-0.294	0.058	-5.107	0.000	***
TMS1	-0.070	0.203	-0.343	0.734	
anteriority1:laterality1	-0.148	0.058	-2.561	0.010	*
anteriority1:SEM1	0.136	0.058	2.358	0.018	*
SEM1:TMS1	-0.308	0.058	-5.329	0.000	***
anteriority1:TMS1	-0.211	0.058	-3.654	0.000	***
laterality1:TMS1	0.140	0.058	2.433	0.015	*
SEM1:laterality1	-0.014	0.058	-0.251	0.802	
anteriority1:SEM1:TMS1	0.036	0.058	0.631	0.528	
anteriority1:laterality1:TMS1	-0.016	0.058	-0.278	0.781	
SEM1:laterality1:TMS1	-0.041	0.058	-0.717	0.474	
anteriority1:SEM1:laterality1	0.027	0.058	0.469	0.639	
anteriority1:SEM1:laterality1:TMS1	-0.032	0.058	-0.549	0.583	

Pairwise contrasts

Anteriority (Contrast)	Estimate	SE	t	p	
Anterior (low - high)	-1.38	0.339	-4.081	0.000	***
Posterior (low - high)	-1.93	0.339	-5.683	0.000	***

TMS condition (Contrast)	Estimate	SE	t	p	
Sham2 (low - high)	-2.27	0.339	-6.691	0.000	***
pSTG/STS (low - high)	-1.04	0.339	-3.070	0.003	**

Model formula: MEAN $\sim$ 1 + anteriority + SEM + laterality + anteriority:laterality + anteriority:SEM + TMS + SEM:TMS + anteriority:TMS + laterality:TMS + anteriority:SEM:TMS + anteriority:laterality:TMS + SEM:laterality + SEM:laterality:TMS + anteriority:SEM:laterality + anteriority:SEM:laterality:TMS + (1 + TMS + SEM | Participant) + (1 + SEM + TMS | Item)

Sum-coding: TMS (-1 = Sham2, 1 = pSTG/STS); SEM (-1 = low cloze, 1 = high cloze).

Significance codes: *** < 0.001, ** < 0.01, * < 0.05, . < 0.1

Table S12. Experiment 1: The effect of early pSTG/STS-rTMS on the N400 at the verb position

Linear Mixed-Effects Model (REML)

Fixed Effects	Estimate	SE	t	p	
(Intercept)	0.512	0.325	1.576	0.125	
TMS1	2.066	0.221	9.339	0.000	***
anteriority1	0.345	0.189	1.822	0.081	.
SEM1	-0.282	0.143	-1.979	0.060	.
laterality1	-0.224	0.065	-3.421	0.001	***
TMS1:anteriority1	0.381	0.154	2.482	0.021	*
TMS1:laterality1	-0.166	0.065	-2.534	0.011	*
TMS1:SEM1	0.148	0.066	2.263	0.024	*
SEM1:laterality1	0.078	0.065	1.199	0.231	
anteriority1:laterality1	-0.065	0.065	-0.986	0.324	
anteriority1:SEM1	-0.037	0.065	-0.567	0.571	
TMS1:anteriority1:laterality1	-0.042	0.065	-0.640	0.522	
TMS1:anteriority1:SEM1	0.067	0.065	1.022	0.307	
anteriority1:SEM1:laterality1	-0.030	0.065	-0.461	0.645	
TMS1:SEM1:laterality1	0.025	0.065	0.382	0.702	
TMS1:anteriority1:SEM1:laterality1	0.043	0.065	0.650	0.516	

Pairwise contrasts

TMS condition (Contrast)	Estimate	SE	t	p	
Sham2 (Low - High)	0.861	0.314	2.740	0.010	*
pSTG/STS (Low - High)	0.268	0.314	0.855	0.399	

Model formula: MEAN $\sim$ 1 + TMS + anteriority + TMS:anteriority + SEM + laterality + TMS:laterality + TMS:SEM + SEM:laterality + anteriority:laterality + TMS:anteriority:laterality + anteriority:SEM + TMS:anteriority:SEM + anteriority:SEM:laterality + TMS:SEM:laterality + TMS:anteriority:SEM:laterality + (1 + TMS + anteriority + SEM + TMS:anteriority | Participant) + (1 | Item)

Sum-coding: TMS (-1 = Sham2, 1 = pSTG/STS); SEM (-1 = low cloze, 1 = high cloze).

Significance codes: *** < 0.001, ** < 0.01, * < 0.05, . < 0.1

Table S13. Experiment 1: The effect of early pIFG-rTMS on the N400 at the noun position

Linear Mixed-Effects Model (REML)

Fixed Effects	Estimate	SE	t	p	
(Intercept)	-1.047	0.319	-3.283	0.003	**
SEM1	0.906	0.117	7.748	0.000	***
anteriority1	0.877	0.166	5.284	0.000	***
laterality1	-0.404	0.058	-6.967	0.000	***
TMS1	-0.040	0.190	-0.209	0.836	
anteriority1:laterality1	-0.185	0.058	-3.189	0.001	**
SEM1:anteriority1	0.112	0.058	1.926	0.054	.
SEM1:laterality1	0.078	0.058	1.341	0.180	
anteriority1:TMS1	-0.166	0.058	-2.869	0.004	***
SEM1:TMS1	0.025	0.058	0.427	0.669	
laterality1:TMS1	-0.024	0.058	-0.419	0.675	
SEM1:anteriority1:TMS1	-0.024	0.058	-0.409	0.683	
anteriority1:laterality1:TMS1	-0.034	0.058	-0.580	0.562	
SEM1:anteriority1:laterality1	0.011	0.058	0.182	0.856	
SEM1:laterality1:TMS1	0.005	0.058	0.087	0.930	
SEM1:anteriority1:laterality1:TMS1	0.004	0.058	0.076	0.940	

Model formula: MEAN $\sim$ 1 + SEM + anteriority + laterality + anteriority:laterality + SEM:anteriority + SEM:laterality + TMS + anteriority:TMS + SEM:TMS + SEM:anteriority:TMS + laterality:TMS + anteriority:laterality:TMS + SEM:anteriority:laterality + SEM:laterality:TMS + SEM:anteriority:laterality:TMS + (1 + TMS + anteriority | Participant) + (1 + SEM | Item)

Sum-coding: TMS (-1 = Sham2, 1 = pIFG); SEM (-1 = low cloze, 1 = high cloze).

Significance codes: *** < 0.001, ** < 0.01, * < 0.05, . < 0.1

Table S14. Experiment 1: The effect of early pIFG-rTMS on the N400 at verb position

Linear Mixed-Effects Model (REML)

Fixed Effects	Estimate	SE	t	p	
(Intercept)	0.651	0.272	2.395	0.025	*
TMS1	2.010	0.264	7.620	0.000	***
SEM1	-0.198	0.064	-3.084	0.002	**
anteriority1	0.179	0.183	0.982	0.337	
laterality1	0.024	0.064	0.374	0.708	
TMS1:anteriority1	0.417	0.064	6.499	0.000	***
TMS1:SEM1	0.084	0.064	1.315	0.189	
SEM1:laterality1	-0.055	0.064	-0.859	0.390	
anteriority1:laterality1	-0.054	0.064	-0.835	0.404	
TMS1:laterality1	-0.027	0.064	-0.423	0.672	
SEM1:anteriority1	0.014	0.064	0.217	0.828	
TMS1:SEM1:laterality1	-0.022	0.064	-0.347	0.729	
TMS1:SEM1:anteriority1	0.028	0.064	0.436	0.663	
SEM1:anteriority1:laterality1	0.010	0.064	0.151	0.880	
TMS1:anteriority1:laterality1	0.007	0.064	0.114	0.909	
TMS1:SEM1:anteriority1:laterality1	-0.019	0.064	-0.303	0.762	

Model formula: MEAN $\sim$ 1 + TMS + SEM + anteriority + TMS:anteriority + TMS:SEM + laterality + SEM:laterality + anteriority:laterality + TMS:laterality + TMS:SEM:laterality + SEM:anteriority + TMS:SEM:anteriority + SEM:anteriority:laterality + TMS:anteriority:laterality + TMS:SEM:anteriority:laterality + (1 + TMS + anteriority | Participant)

Sum-coding: TMS (-1 = Sham2, 1 = pIFG); SEM (-1 = low cloze, 1 = high cloze).

Significance codes: *** < 0.001, ** < 0.01, * < 0.05, . < 0.1

Table S15. Experiment 2: The effect of AG cTBS on the N400 at the noun position

Linear Mixed-Effects Model (REML)

Fixed Effects	Estimate	SE	t	p	
(Intercept)	-1.139	0.326	-3.493	0.002	**
SEM1	0.992	0.164	6.061	0.000	***
anteriority1	0.757	0.113	6.697	0.000	***
laterality1	-0.225	0.058	-3.886	0.000	***
TMS1	0.131	0.058	2.251	0.024	*
SEM1:anteriority1	0.138	0.058	2.380	0.017	*
anteriority1:TMS1	0.119	0.058	2.056	0.040	*
anteriority1:laterality1	-0.099	0.058	-1.709	0.087	.
laterality1:TMS1	-0.081	0.058	-1.402	0.161	
SEM1:TMS1	-0.080	0.058	-1.382	0.167	
SEM1:laterality1	-0.010	0.058	-0.164	0.870	
anteriority1:laterality1:TMS1	-0.011	0.058	-0.190	0.849	
SEM1:laterality1:TMS1	0.051	0.058	0.885	0.376	
SEM1:anteriority1:laterality1	-0.017	0.058	-0.302	0.763	
SEM1:anteriority1:TMS1	-0.007	0.058	-0.125	0.901	
SEM1:anteriority1:laterality1:TMS1	0.001	0.058	0.017	0.986	

Pairwise Contrasts

Anteriority (Contrast)	Estimate	SE	t	p	
Anterior (Low - High)	-1.71	0.347	-4.919	0.000	***
Posterior (Low - High)	-2.26	0.347	-6.508	0.000	***

Model formula: MEAN $\sim$ 1 + SEM + anteriority + laterality + SEM:anteriority + TMS + anteriority:TMS + anteriority:laterality + laterality:TMS + SEM:TMS + anteriority:laterality:TMS + SEM:laterality + SEM:laterality:TMS + SEM:anteriority:laterality + SEM:anteriority:TMS + SEM:anteriority:laterality:TMS + (1 + anteriority + SEM | Participant) + (1 + SEM | Item)

Sum-coding: TMS (-1 = Sham2, 1 = pIFG); SEM (-1 = low cloze, 1 = high cloze).

Significance codes: *** < 0.001, ** < 0.01, * < 0.05, . < 0.1

Table S16. Experiment 2: The effect of AG cTBS on the N400 at the verb position

Linear Mixed-Effects Model (REML)

Fixed Effects	Estimate	SE	t	p	
(Intercept)	-1.050	0.285	-3.688	0.001	***
anteriority1	-0.598	0.179	-3.340	0.003	**
SEM1	-0.327	0.140	-2.342	0.024	*
laterality1	-0.057	0.062	-0.910	0.363	
TMS1	0.025	0.093	0.263	0.795	
anteriority1:laterality1	0.057	0.062	0.915	0.360	
anteriority1:SEM1	-0.040	0.062	-0.636	0.525	
SEM1:laterality1	0.021	0.062	0.338	0.735	
anteriority1:TMS1	0.119	0.062	1.907	0.057	.
SEM1:TMS1	-0.081	0.062	-1.304	0.192	
laterality1:TMS1	-0.029	0.062	-0.462	0.644	
anteriority1:SEM1:laterality1	0.030	0.062	0.481	0.631	
anteriority1:laterality1:TMS1	0.037	0.062	0.588	0.557	
anteriority1:SEM1:TMS1	0.026	0.062	0.414	0.679	
SEM1:laterality1:TMS1	-0.005	0.062	-0.081	0.936	
anteriority1:SEM1:laterality1:TMS1	-0.012	0.062	-0.199	0.842	

Model formula: MEAN $\sim$ 1 + anteriority + SEM + laterality + anteriority:laterality + anteriority:SEM + SEM:laterality + anteriority:SEM:laterality + TMS + anteriority:TMS + SEM:TMS + laterality:TMS + anteriority:laterality:TMS + anteriority:SEM:TMS + SEM:laterality:TMS + anteriority:SEM:laterality:TMS + (1 + anteriority + TMS | Participant) + (1 + SEM | Item)

Sum-coding: TMS (-1 = Sham1, 1 = Sham2); SEM (-1 = low cloze, 1 = high cloze).

Significance codes: *** < 0.001, ** < 0.01, * < 0.05, . < 0.1

Table S17. Experiment 2: The effect of midtime pIFG-rTMS on the N400 at noun position

Linear Mixed-Effects Model (REML)

Fixed Effects	Estimate	SE	t	p	
(Intercept)	-1.163	0.345	-3.371	0.002	**
SEM1	0.772	0.155	4.978	0.000	***
anteriority1	0.690	0.122	5.677	0.000	***
laterality1	-0.172	0.058	-2.995	0.003	**
TMS1	-0.172	0.058	-2.988	0.003	**
anteriority1:TMS1	-0.185	0.058	-3.215	0.001	**
SEM1:anteriority1	0.167	0.058	2.901	0.004	**
SEM1:TMS1	-0.127	0.058	-2.201	0.028	*
laterality1:TMS1	0.134	0.058	2.329	0.020	*
anteriority1:laterality1	-0.092	0.058	-1.592	0.111	
SEM1:laterality1	0.007	0.058	0.127	0.899	
SEM1:anteriority1:TMS1	0.035	0.058	0.612	0.541	
anteriority1:laterality1:TMS1	0.018	0.058	0.320	0.749	
SEM1:laterality1:TMS1	-0.034	0.058	-0.598	0.550	
SEM1:anteriority1:laterality1	0.003	0.058	0.059	0.953	
SEM1:anteriority1:laterality1:TMS1	0.020	0.058	0.345	0.730	

Pairwise Contrasts

Anteriority (Contrast)	Estimate	SE	t	p	
Anterior (Low - High)	-1.21	0.331	-3.657	0.001	**
Posterior (Low - High)	-1.88	0.331	-5.676	0.000	***

TMS condition (Contrast)	Estimate	SE	t	p	
Sham2 (Low - High)	-1.80	0.331	-5.431	0.000	***
pIFG (Low - High)	-1.29	0.331	-3.901	0.000	***

Model formula: MEAN $\sim$ 1 + SEM + anteriority + laterality + TMS + anteriority:TMS + SEM:anteriority + SEM:TMS + laterality:TMS + anteriority:laterality + SEM:anteriority:TMS + anteriority:laterality:TMS + SEM:laterality + SEM:laterality:TMS + SEM:anteriority:laterality + SEM:anteriority:laterality:TMS + (1 + SEM + anteriority | Participant) + (1 | Item)

Sum-coding: TMS (-1 = Sham2, 1 = pIFG); SEM (-1 = low cloze, 1 = high cloze).

Significance codes: *** < 0.001, ** < 0.01, * < 0.05, . < 0.1

Table S18. Experiment 3: The effect of midtime pIFG-rTMS on the N400 at the noun position

Linear Mixed-Effects Model (REML)

Fixed Effects	Estimate	SE	t	p	
(Intercept)	-1.564	0.235	-6.651	0.000	***
TMS1	0.417	0.140	2.973	0.005	**
SEM1	0.277	0.115	2.400	0.021	*
laterality1	-0.213	0.062	-3.455	0.001	***
anteriority1	0.003	0.073	0.039	0.969	
TMS1:SEM1	-0.133	0.062	-2.161	0.031	*
SEM1:laterality1	-0.069	0.062	-1.126	0.260	
TMS1:laterality1	-0.011	0.062	-0.185	0.853	
SEM1:anteriority1	0.116	0.062	1.877	0.060	
laterality1:anteriority1	0.035	0.062	0.569	0.570	
TMS1:anteriority1	0.028	0.062	0.448	0.655	
TMS1:SEM1:laterality1	0.019	0.062	0.305	0.761	
TMS1:SEM1:anteriority1	0.043	0.062	0.694	0.488	
TMS1:laterality1:anteriority1	0.031	0.062	0.503	0.615	
SEM1:laterality1:anteriority1	0.009	0.062	0.139	0.889	
TMS1:SEM1:laterality1:anteriority1	-0.018	0.062	-0.289	0.773	

Pairwise Contrasts

TMS Condition (Contrast)	Estimate	SE	t	p	
Vertex (Low - High)	-0.1211	0.0386	-3.137	0.0026	**
pIFG (Low - High)	-0.0423	0.0386	-1.096	0.2774	

Model formula: MEAN $\sim$ 1 + TMS + SEM + laterality + TMS:SEM + SEM:laterality + TMS:laterality + TMS:SEM:laterality + anteriority + SEM:anteriority + laterality:anteriority + TMS:anteriority + TMS:SEM:anteriority + TMS:laterality:anteriority + SEM:laterality:anteriority + TMS:SEM:laterality:anteriority + (1 + TMS | Participant) + (1 + TMS + anteriority + SEM | Item)

Sum-coding: TMS (-1 = Sham2, 1 = pIFG); SEM (-1 = low cloze, 1 = high cloze).

Significance codes: *** < 0.001, ** < 0.01, * < 0.05, . < 0.1

Table S19. Experiment 3: The effect of midtime pSTG/STS-rTMS on the N400 at the noun position

Linear Mixed-Effects Model (REML)

Fixed Effects	Estimate	SE	t	p	
(Intercept)	-1.849	0.197	-9.371	0.000	***
SEM1	0.320	0.063	5.082	0.000	***
laterality1	-0.177	0.063	-2.820	0.005	**
TMS1	0.128	0.212	0.604	0.551	
anteriority1	0.007	0.063	0.106	0.916	
SEM1:TMS1	-0.094	0.063	-1.495	0.135	
SEM1:laterality1	-0.038	0.063	-0.600	0.549	
laterality1:TMS1	0.024	0.063	0.383	0.702	
SEM1:anteriority1	0.132	0.063	2.103	0.036	*
TMS1:anteriority1	0.028	0.063	0.452	0.651	
laterality1:anteriority1	-0.001	0.063	-0.016	0.987	
SEM1:laterality1:TMS1	0.050	0.063	0.801	0.423	
SEM1:TMS1:anteriority1	0.059	0.063	0.935	0.350	
SEM1:laterality1:anteriority1	0.009	0.063	0.143	0.887	
laterality1:TMS1:anteriority1	-0.005	0.063	-0.080	0.936	
SEM1:laterality1:TMS1:anteriority1	-0.017	0.063	-0.277	0.782	

Model formula: MEAN $\sim$ 1 + SEM + laterality + TMS + SEM:TMS + SEM:laterality + laterality:TMS + SEM:laterality:TMS + anteriority + SEM:anteriority + TMS:anteriority + SEM:TMS:anteriority + laterality:anteriority + SEM:laterality:anteriority + laterality:TMS:anteriority + SEM:laterality:TMS:anteriority + (1 + TMS | Item) + (1 + TMS | Participant)

Sum-coding: TMS (-1 = Sham2, 1 = pSTG/STS); SEM (-1 = low cloze, 1 = high cloze).

Significance codes: *** < 0.001, ** < 0.01, * < 0.05, . < 0.1

Table S20. Experiment 3: The effect of late pSTG/STS-rTMS on the N400 at the noun position

Linear Mixed-Effects Model (REML)

Fixed Effects	Estimate	SE	t	p	
(Intercept)	-1.982	0.286	-6.922	0.000	***
SEM1	0.204	0.065	3.130	0.002	**
TMS1	-0.114	0.275	-0.415	0.681	
laterality1	-0.059	0.094	-0.632	0.534	
anteriority1	-0.055	0.122	-0.449	0.656	
SEM1:TMS1	-0.158	0.065	-2.429	0.015	*
TMS1:anteriority1	-0.103	0.065	-1.580	0.114	
SEM1:anteriority1	0.057	0.065	0.884	0.377	
SEM1:laterality1	0.033	0.065	0.511	0.609	
laterality1:anteriority1	0.030	0.065	0.457	0.647	
TMS1:laterality1	0.010	0.065	0.146	0.884	
SEM1:TMS1:anteriority1	-0.062	0.065	-0.951	0.342	
TMS1:laterality1:anteriority1	0.026	0.065	0.393	0.694	
SEM1:TMS1:laterality1	0.006	0.065	0.096	0.924	
SEM1:laterality1:anteriority1	0.002	0.065	0.031	0.976	
SEM1:TMS1:laterality1:anteriority1	-0.005	0.065	-0.076	0.939	

Pairwise Contrasts

TMS Condition (Contrast)	Estimate	SE	t	p	
Vertex (Low - High)	-0.7232	0.184	-3.929	0.0001	***
pIFG (Low - High)	-0.0913	0.184	-0.497	0.6195	

Model formula: MEAN $\sim$ 1 + SEM + TMS + SEM:TMS + laterality + anteriority + TMS:anteriority + SEM:anteriority + SEM:TMS:anteriority + SEM:laterality + laterality:anteriority + TMS:laterality + TMS:laterality:anteriority + SEM:TMS:laterality + SEM:laterality:anteriority + SEM:TMS:laterality:anteriority + (1 + TMS + anteriority + laterality | Participant) + (1 + TMS + anteriority | Item)

Sum-coding: TMS (-1 = Sham2, 1 = pSTG/STS); SEM = (-1 = low cloze, 1 = high cloze).

Significance codes: *** < 0.001, ** < 0.01, * < 0.05, . < 0.1

Table S21. Experiment 3: The effect of late pIFG-rTMS on the N400 at the noun position

Linear Mixed-Effects Model (REML)

Fixed Effects	Estimate	SE	t	p	
(Intercept)	-1.607	0.260	-6.188	0.000	***
SEM1	0.270	0.062	4.321	0.000	***
TMS1	0.262	0.200	1.308	0.199	
laterality1	-0.163	0.062	-2.611	0.009	**
anteriority1	-0.078	0.094	-0.829	0.416	
TMS1:laterality1	-0.095	0.062	-1.516	0.129	
SEM1:TMS1	-0.092	0.062	-1.472	0.141	
TMS1:anteriority1	-0.125	0.062	-2.010	0.044	*
SEM1:anteriority1	0.077	0.062	1.237	0.216	
laterality1:anteriority1	0.038	0.062	0.607	0.544	
SEM1:laterality1	0.032	0.062	0.507	0.612	
SEM1:TMS1:anteriority1	-0.041	0.062	-0.658	0.511	
TMS1:laterality1:anteriority1	0.034	0.062	0.540	0.589	
SEM1:TMS1:laterality1	0.004	0.062	0.063	0.949	
SEM1:laterality1:anteriority1	-0.001	0.062	-0.018	0.985	
SEM1:TMS1:laterality1:anteriority1	-0.008	0.062	-0.130	0.897	

Model formula: MEAN $\sim$ 1 + SEM + TMS + laterality + TMS:laterality + SEM:TMS + anteriority + TMS:anteriority + SEM:anteriority + SEM:TMS:anteriority + laterality:anteriority + TMS:laterality:anteriority + SEM:laterality + SEM:TMS:laterality + SEM:laterality:anteriority + SEM:TMS:laterality:anteriority + (1 + TMS + anteriority | Participant) + (1 + TMS | Item)

Sum-coding: TMS (-1 = Sham2, 1 = pIFG); SEM (-1 = low cloze, 1 = high cloze).

Significance codes: *** < 0.001, ** < 0.01, * < 0.05, . < 0.1

Table S22. EXTRA: Experiment 3 (middle time-window): pIFG versus pSTG/STS rTMS on the N400 at the noun position

Linear Mixed-Effects Model (REML)

Fixed Effects	Estimate	SE	t	p	
(Intercept)	-1.436	0.176	-8.163	0.000	***
TMS1	-0.287	0.199	-1.445	0.160	
SEM1	0.189	0.052	3.665	0.000	***
laterality1	-0.189	0.051	-3.663	0.000	***
anteriority1	0.036	0.102	0.350	0.730	
TMS1:SEM1	0.036	0.052	0.698	0.485	
TMS1:laterality1	0.035	0.051	0.689	0.491	
SEM1:anteriority1	0.175	0.051	3.406	0.001	***
laterality1:anteriority1	0.030	0.051	0.582	0.561	
SEM1:laterality1	-0.019	0.051	-0.368	0.713	
TMS1:anteriority1	0.000	0.051	-0.001	0.999	
TMS1:SEM1:laterality1	0.032	0.051	0.614	0.540	
SEM1:laterality1:anteriority1	-0.009	0.051	-0.171	0.864	
TMS1:laterality1:anteriority1	-0.036	0.051	-0.700	0.484	
TMS1:SEM1:anteriority1	0.017	0.051	0.321	0.748	
TMS1:SEM1:laterality1:anteriority1	0.000	0.051	0.008	0.994	

Model formula: MEAN $\sim$ 1 + SEM + TMS + laterality + SEM:TMS + TMS:laterality + anteriority + SEM:anteriority + laterality:anteriority + SEM:laterality + SEM:TMS:laterality + SEM:laterality:anteriority + SEM:TMS:laterality:anteriority + TMS:anteriority + TMS:laterality:anteriority + TMS:SEM:anteriority + TMS:SEM:laterality:anteriority + (1 + TMS + anteriority | Participant) + (1 + TMS | Item)

Sum-coding: TMS (-1 = pIFG, 1 = pSTG); SEM (-1 = low cloze, 1 = high cloze).

Significance codes: *** < 0.001, ** < 0.01, * < 0.05, . < 0.1

Table S23. EXTRA: Experiment 3 (late time-window): pIFG versus pSTG/STS rTMS on the N400 at the noun position

Linear Mixed-Effects Model (REML)

Fixed Effects	Estimate	SE	t	p	
(Intercept)	-1.724	0.299	-5.766	0.000	***
TMS1	-0.378	0.255	-1.485	0.151	
anteriority1	-0.183	0.137	-1.335	0.192	
laterality1	-0.154	0.138	-1.119	0.275	
SEM1	0.112	0.054	2.084	0.037	*
TMS1:laterality1	0.105	0.054	1.952	0.051	.
TMS1:SEM1	-0.066	0.054	-1.233	0.217	
anteriority1:laterality1	0.063	0.054	1.178	0.239	
laterality1:SEM1	0.039	0.054	0.717	0.473	
TMS1:anteriority1	0.027	0.118	0.233	0.818	
anteriority1:SEM1	0.018	0.054	0.327	0.743	
TMS1:anteriority1:SEM1	-0.020	0.054	-0.379	0.704	
TMS1:anteriority1:laterality1	-0.008	0.054	-0.151	0.880	
anteriority1:laterality1:SEM1	-0.006	0.054	-0.113	0.910	
TMS1:laterality1:SEM1	0.002	0.054	0.038	0.970	
TMS1:anteriority1:laterality1:SEM1	0.003	0.054	0.058	0.954	

Model formula: MEAN $\sim$ 1 + TMS + anteriority + laterality + SEM + TMS:laterality + TMS:SEM + anteriority:laterality + laterality:SEM + TMS:anteriority + anteriority:SEM + TMS:anteriority:SEM + TMS:anteriority:laterality + anteriority:laterality:SEM + TMS:laterality:SEM + TMS:anteriority:laterality:SEM + (1 + TMS + laterality + anteriority + TMS:anteriority | Participant) + (1 + anteriority | Item)

Sum-coding: TMS (-1 = pIFG, 1 = pSTG); SEM (-1 = low cloze, 1 = high cloze).

Significance codes: *** < 0.001, ** < 0.01, * < 0.05, . < 0.1

References

1. J Obleser, SA Kotz, Expectancy Constraints in Degraded Speech Modulate the Language Comprehension Network. *Cereb. Cortex* **20**, 633–640 (2010).
2. MA Mayka, DM Corcos, SE Leurgans, DE Vaillancourt, Three-dimensional locations and boundaries of motor and premotor cortices as defined by functional brain imaging: A meta-analysis. *NeuroImage* **31**, 1453–1474 (2006).
3. LOH Kroczek, TC Gunter, AU Rysop, AD Friederici, G Hartwigsen, Contributions of left frontal and temporal cortex to sentence comprehension: Evidence from simultaneous TMS-EEG. *Cortex* **115**, 86–98 (2019).
4. P Kuhnke, L Meyer, AD Friederici, G Hartwigsen, Left posterior inferior frontal gyrus is causally involved in reordering during sentence processing. *NeuroImage* **148**, 254–263 (2017).
5. MG Stokes, et al., Simple metric for scaling motor threshold based on scalp-cortex distance: Application to studies using transcranial magnetic stimulation. *J. Neurophysiol.* **94**, 4520–4527 (2005).
6. V Piai, L Nieberlein, G Hartwigsen, Effects of transcranial magnetic stimulation over the left posterior superior temporal gyrus on picture-word interference. *PLoS ONE* **15**, 1–18 (2020).
7. G Hartwigsen, T Golombek, J Obleser, Repetitive transcranial magnetic stimulation over left angular gyrus modulates the predictability gain in degraded speech comprehension. *Cortex* **68**, 100–110 (2015).
8. G Hartwigsen, et al., Dissociating parieto-frontal networks for phonological and semantic word decisions: a condition-and-perturb TMS study. *Cereb. cortex* **26**, 2590–2601 (2016).
9. G Hartwigsen, et al., The right posterior inferior frontal gyrus contributes to phonological word decisions in the healthy brain: Evidence from dual-site TMS. *Neuropsychologia* **48**, 3155–3163 (2010).
10. G Hartwigsen, et al., Left dorsal premotor cortex and supramarginal gyrus complement each other during rapid action reprogramming. *J. Neurosci.* **32**, 16162–16171 (2012).
11. P Kuhnke, et al., Left posterior inferior parietal cortex causally supports the retrieval of action knowledge. *NeuroImage* **219**, 117041 (2020).
